# Exploring the Complex Relationship between Biological Aging and Cancer in a Prospective Italian Population Cohort

**DOI:** 10.14336/AD.2025.0204

**Published:** 2026-05-27

**Authors:** Martina Morelli, Antonietta Pepe, Simona Costanzo, Teresa Panzera, Sara Magnacca, Amalia De Curtis, Maria Loreto Muñoz Venegas, Chiara Cerletti, Maria Benedetta Donati, Giovanni de Gaetano, Licia Iacoviello, Alessandro Gialluisi, on-behalf-of the Moli-sani Study Investigators

**Affiliations:** ^1^Research Unit of Epidemiology and Prevention, IRCCS Neuromed, Pozzilli, Italy.; ^2^Department of Medicine and Surgery, LUM University, Casamassima, Italy.; ^3^Department of Medicine and Surgery, Research Center in Epidemiology and Preventive Medicine (EPIMED), University of Insubria, Varese, Italy

**Keywords:** biological aging, phenotypic aging, risk factor, cancer mortality, cancer risk, inflammation, chronic diseases

## Abstract

Cancer is often associated with age-related chronic conditions; however, the role of biological aging as a potential risk factor for cancer remains unclear and largely unexplored. To clarify this link, we tested the influence of two biological aging measures in an Italian prospective population cohort, the Moli-sani study (N=24,325; age ≥35 years; 51.9% women). For each participant these were based on the difference between first and a second-generation blood-based biological age estimates (BloodAge and PhenoAge) and chronological age. The resulting biological aging acceleration measures (BloodAgeAcc and PhenoAgeAcc) were tested for association with cancer mortality, first hospitalization and incident fatal/non-fatal cases, using multivariable Cox proportional hazard models. We analyzed 22,985 apparently cancer-free participants with mortality data available (median follow-up 13.1 years) and found independent increases in mortality rates per one-year increase in PhenoAgeAcc and BloodAgeAcc. Additionally, statistically significant increased risk of cancer hospitalizations was observed for PhenoAgeAcc. The analysis of incident cancers for different body sites identified both increased and decreased risks associated with biological aging acceleration. BloodAgeAcc was indeed weakly associated with a reduced risk of both breast and prostate cancer, but with an increased risk of pancreatic cancer, while PhenoAgeAcc was associated with an increased risk of lung and renal cancer. Our findings suggest that biological aging acceleration may differently impact cancer-related risks, with protective effects for some cancers versus increased risks for others. Further independent cohort studies are needed to clarify the translational clinical impact of these findings.

## INTRODUCTION

Aging is closely linked to cancer and some of the proposed characteristics of aging, such as genomic instability, cellular senescence, autophagy and epigenetic alterations also partially overlap with cancer [1]. Initially, cancer and aging may be considered as tagging opposite processes where cancer is taken as a consequence of an aberrant gain of cellular fitness which results in uncontrolled cell proliferation, while aging is characterized by a loss of fitness and cell death [2]. However, cancer and aging share common origins, namely the time-dependent accumulation of cellular damage which is widely considered as the general cause of aging, but can occasionally provide aberrant advantages to certain cells which could eventually generate neoplastic lesions [3–5]. Therefore, cancer and aging can be regarded as two faces of the same coin [2].

More so, most cancers have been associated with chronological age (CA) and aging in general [6]. Besides CA, several indices of biological age (BA) – namely measures representing the actual age of an organism - have been developed in the last decades through different computational approaches, including supervised machine learning algorithms and different sources of clinical and biological data, and have been reported to predict mortality and morbidity even better than CA [7]. Biological aging of a subject can be measured through the difference between BA and CA (hereafter referred to as Δage, [8]), which can result in biological aging acceleration (Δage > 0), deceleration (Δage < 0), or in a biological age comparable to chronological age (Δage ~ 0). From a biological perspective, aging is related to accumulated molecular and cellular damage to body systems over the life course, leading to a loss of reserve and capacity to respond to challenges, vulnerability to chronic diseases, deterioration in function and ultimately to death [9]. Although CA is an obvious predictor of mortality, biological aging captures the heterogeneity of risks among elderly individuals [10]. In fact, BA measures frequently integrate data from multiple biological markers, potentially offering a more accurate reflection of an individual's physiological state and associated risks of age-related morbidities and mortality [11]. Consequently, measures of discrepancy between BA and CA (also known as *biological aging clocks*) have been evaluated for their predictive capacity of several age-related clinical events and health conditions. These studies demonstrated that such aging clocks predict mortality [12] or the development of health conditions like cardiovascular disease [13], diabetes [14] and cancer [15], often better than CA does. Still, the evidence resulting from epidemiological and statistical genetic studies on the link between biological aging acceleration and incident risk of cancer is controversial, with some studies reporting an increased risk of all cancers [15], as well as lung [16], breast [17, 18] and colorectal cancer [17, 19, 16], while other studies report a protective association (e.g. with the incident risk of prostate cancer [17]).

Moreover, there is a scarcity of studies on the association between composite aging clocks - especially those based on clinical biomarkers commonly available in the medical routine - and the incident risk of cancer. Based on this, the aim of our study was to investigate the relationship between aging clocks based on common blood markers and cancer-related risks in an Italian population cohort. Firstly, we focused on the main clinical events related to cancer such as cancer deaths and hospitalizations, and then on incidents (both fatal and non-fatal) cancer events for specific causes.

## METHODS

### Study Population

The Moli-sani Study is a prospective cohort study established in 2005-2010 with the enrolment of 24,325 men and women (aged ≥35 years) randomly recruited from the general population of Molise, a Southern Mediterranean Italian region, with the purpose of investigating risk factors for chronic disease conditions, including also cardiovascular and cancer diseases. The exclusion criteria at recruitment time were pregnant status; altered understanding capacity or willingness; current poly-traumas or coma; and/or refusal to sign the informed consent form. The Moli-sani study complied with the Declaration of Helsinki and was granted the approval of the Ethics Committee of the Catholic University, Rome, Italy. Details of the study design are available elsewhere [20, 21].

#### Blood biomarkers Protocol

Blood samples were collected between 7:00 and 9:00 AM from participants who had fasted overnight and had abstained from smoking for at least 6 hours. Biochemical analyses were conducted at the centralized Moli-sani laboratory, as previously described [22]. Hemochromo-cytometric analyses were carried out using a cell counter (Coulter HMX, Beckman Coulter, Milan, Italy) within 3 hours from venipuncture. Analyses of creatinine, cystatin C, N-Terminal Pro-B-Type Natriuretic Peptide, testosterone, high sensitivity troponin I and vitamin D were performed within the BiomarCaRE Consortium and have been described in detail elsewhere (see https://cordis.europa.eu/project/id/278913/reporting/it) [23]. A full description of the markers used can be found in Gialluisi et al 2022 [13].

#### Aging Clocks

Within the Moli-sani cohort, we computed two blood-based markers of biological aging, a first-generation marker (i.e. aimed at estimating chronological age of individuals as precisely as possible, hereafter called blood age or BloodAge) and a second-generation marker (aimed at estimating clinical risks related to aging, like mortality, hereafter called phenotypic age or PhenoAge). The methods used for the computation of these markers are explained below, with further details found in the original manuscripts [24, 13, 25]. To deploy these algorithms, dedicated scripts and packages were used in R versions 3.9 to 4.2.2 (depending on the packages and on the time of analyses; https://cordis.europa.eu/project/id/278913/reporting/it).

#### BloodAge

To compute BloodAge, we used a Deep Neural Network (DNN), through the Keras R-package version 2.4.0 (https://cran.r-project.org/web/packages/keras/index.html).

From the initial 24,325 participants, 23,858 samples passed our quality control (QC) checks, after re-moving subjects reporting non-Italian ancestry and/or non-fasting state at the time of blood drawn. Blood markers measured were filtered out if they represented composite biomarkers (including total cholesterol, plateletcrit, haematocrit and mean corpuscular hemoglobin) to avoid collinearity. Unreliable values of some blood markers - i.e., leukocyte counts whose fractions summed <99% or >101% - were set to missing and then imputed through either a k-nearest neighbour (kNN) algorithm or a minimum/maximum imputation (when measures were below/above the detection limits).

**Figure 1.**
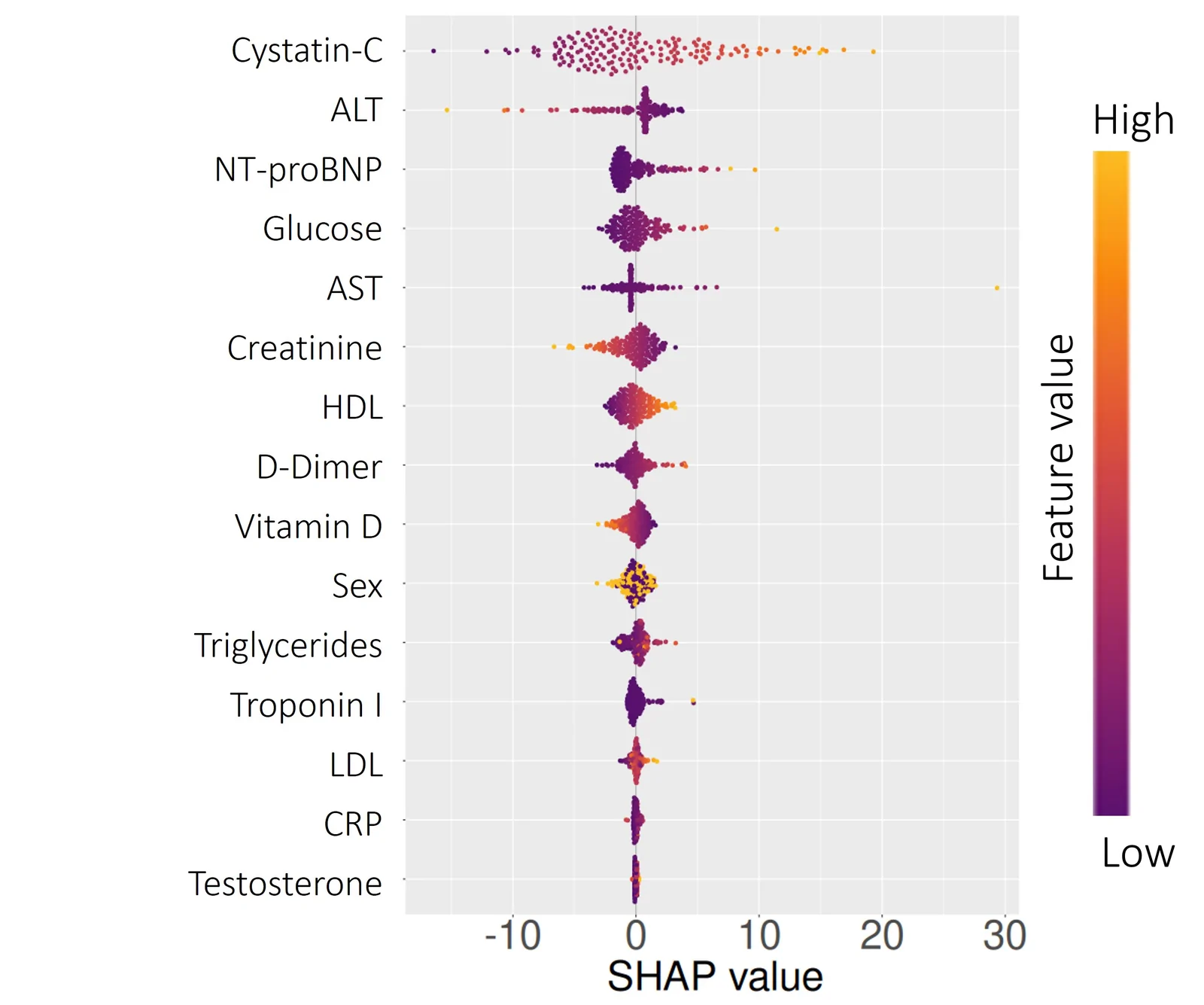
**Most influential features in the algorithm used for BloodAge computation, ranked by mean SHAP (SHapley Additive exPlanations) values.** The beeswarm plot provides information on how model’s features impact its predictions in terms of strength (absolute SHAP values) and direction (sign of the SHAP values) of the effect. Features with positive SHAP values positively impact the prediction, while those with negative values have a negative effect. Higher absolute SHAP values indicated higher (negative or positive) impact on prediction. The strength of colour bar indicates the features’ values: relatively high values are depicted in yellow while relatively low values are depicted in violet. For sex, yellow indicates male and violet indicates female. In our model, high values of Cystatin-C, NT-proBNP, Glucose, HDL, D-Dimer have positive SHAP values and are thus associated to higher predicted BloodAge, that is faster aging. On the other hand, high values of ALT, Creatinine and Vitamin-D are associated to negative SHAP values and thus to lower predicted BloodAge. Other features have a smaller feature effect onto the predicted BloodAge. Abbreviations: NT-proBNP: NT-proB-type Natriuretic Peptide; AST: aspartate transaminase; ALT: alanine aminotransferase; HDL/LDL: high and low density lipoprotein cholesterol; CRP: high sensitivity C-reactive protein.

This left 36 circulating markers available for analysis, including lipid biomarkers (triglycerides, high and low density lipoprotein (HDL and LDL) cholesterol, lipoprotein A and apolipoprotein A1 and B); markers of glucose metabolism (glucose, C-peptide and insulin); liver enzymes (aspartate transaminase (AST) and alanine aminotransferase (ALT)); cardiac and vascular markers (NT-proB-type Natriuretic Peptide and high-sensitivity cardiac troponin I); other hormones (testosterone and vitamin D); haemostasis markers such as D-dimer; renal markers (uric acid, albumin, creatinine, cystatin-C); inflammation markers like high sensitivity C-reactive protein (CRP); common haemochrome markers (red blood cell count and distribution width, haemoglobin levels, mean corpuscular volume, mean corpuscular haemoglobin concentration, total white blood cells, lymphocytes, monocytes, granulocytes, neutrophils, basophils and eosinophils, platelet count, mean platelet volume and platelet distribution width). These features underwent normalization before analysis and were used as input features of the DNN, along with recruiting centre and sex of each participant, for the prediction of BloodAge using CA as a label. In the original manuscript [13], we split the available dataset passing QC (N=23,858) into a random training and test set (80:20 ratio), then trained the algorithm over 1,000 epochs in the training set and evaluated the accuracy in the test set (N = 4,772). Here, to ensure maximum power to our association tests and to allow replicability in other independent cohorts, we exploited the totality of Moli-sani participants passing QC and with blood biomarkers available, computing BloodAge based on the fifteen most influential features in the deployed algorithm. These included cystatin C, NT-proB-type Natriuretic Peptide, aspartate transaminase and alanine aminotransferase, troponin I, creatinine, glucose, sex, high- and low-density lipoprotein cholesterol, triglycerides, D-dimer, vitamin D, testosterone and high sensitivity C-reactive protein levels (see SHapley Additive exPlanations (SHAP) values in Figure 1). The algorithm above has shown comparable performance with the full DNN algorithm in terms of accuracy for CA prediction, and is publicly available for download [13]. We then computed BloodAge and the resulting discrepancy with CA (∆BloodAge = BloodAge–CA) for these participants, which were then used as the population of study for all the downstream analyses.

#### PhenoAge

PhenoAge is conceived as a blood-based biological age measure based on the application of Gompertz survival models to age and nine different circulating biomarkers, including albumin, creatinine, glucose, CRP, mean cell volume (MCV), red cell distribution width (RDW), alkaline phosphatase (ALP), white blood cell count (WBC) and lymphocyte fraction [25]. To compute this, we used the BioAge package (https://github.com/dayoonkwon/BioAge), applying it to all the above-mentioned features except ALP, which was not available in the Moli-sani cohort. The resulting PhenoAge measure was previously reported to have comparable performance in mortality risk prediction and a high correlation with the original PhenoAge measure [13]. The discrepancy between PhenoAge and chronological age (ΔPhenoAge= PhenoAge–CA) was computed for each participant and tested as an additional index of biological aging, based mostly on cellular and immunity-related markers. This measure was reported to outperform ∆BloodAge in predicting all-cause mortality and first hospitalization risk within the Moli-sani cohort – although the two clocks showed independent significant associations with both events [13] – and was preferred over other (e.g. epigenetic) aging clocks due to the large availability of blood marker measures in the study.

#### Incident Cancer Deaths, Hospitalizations and fatal/non-fatal cases

The Moli-sani study cohort was followed-up for cancer deaths, hospitalizations and fatal/non-fatal events from study entry (2005–2010) through 31 December 2020. Fatal and non-fatal cancer events in our dataset were identified through systematic procedures that ensured its accuracy and reliability.

Cancer deaths were ascertained by direct linkage with the regional death register (ReNCaM register) when underlying cause of death presented specific ICD-9 (9th version of the International Classification of Diseases) codes: 140–172 and 174–208.

Cancer hospitalization incidence was defined as the earliest diagnosis of cancer from hospital discharge records. A hospitalization was defined as any length of stay of at least 24h in a hospital or clinic. If a patient was transferred to another hospital or facility, a single hospitalization event was considered. Hospitalizations for rehabilitation and chemotherapy and/or radiotherapy (i.e., elective treatment in a hospital-based unit) were excluded. Primary and secondary diagnoses for hospitalization were coded using ICD-9 codes. A cancer hospitalization was assessed if the primary or the first secondary diagnosis of admission to the hospital were coded as ICD-9: 140–172 and 174–208.

For the most frequent cancer types, similar methods were employed, using regional hospital discharge forms and death registries, along with the consultation of medical records to ensure comprehensive ascertainment of cancer incidence cases. Incident cases of breast (BC), prostate (PC), colorectal (CRC), lung (LC), pancreatic (PNC) and renal (RNC) cancers were ascertained by direct linkage with hospital discharge forms according to the ICD-9-CM code (BC: 174, PC:185, CRC: 153-154, LC:162-163, PNC: 157, RNC: 189). Events were validated through medical records when the cancer was mentioned in the diagnosis and was confirmed by histological reports.

#### Statistical Analysis

All statistical analyses were both performed in R (version 4.2.2; https://www.r-project.org/).

Out of 23,858 subjects for whom BloodAge or PhenoAge clock data were available, 22,985 participants were left for analyses after excluding subjects who reported medical history of cancer at recruitment and subjects for whom cancer event ascertainment was not possible, representing our final analyzed sample.

In these samples, ΔPhenoAge and ΔBloodAge were residualized against CA in univariate linear regressions to remove their partial dependence. The resulting residuals – hereafter called Phenotypic Age acceleration (PhenoAgeAcc) and Blood Age acceleration (BloodAgeAcc) indices - were first tested for association with incident cancer deaths and hospitalizations and, in a second instance, with incident cancer cases for specific causes (BC, CRC, LC, PC, RNC and PNC), both fatal and non-fatal, to clarify the relationship between systemic biological aging and specific cancer risks.

All the associations were tested for both PhenoAgeAcc and BloodAgeAcc, first separately in multivariable Cox proportional hazards (PH) regressions incrementally adjusted for (i) age, sex (Model 1), and (ii) prevalent health conditions like cardiovascular diseases (CVD), type 2 diabetes (T2D), hyperlipidemia and body mass index (BMI) (Model 2). Then, we tested the two aging clocks jointly in a final adjusted Cox PH model (Model 3). For all the covariates except PhenoAgeAcc and BloodAgeAcc, a case-complete approach was used (see Table S1a, b for missing data statistics). Lifestyles were not used as covariates in the main models since they may act as ancestral exposures in the modeled association, influencing the clinical risks analyzed also through increasing the rate of biological aging (i.e. PhenoAgeAcc and BloodAgeAcc). However, we considered it appropriate to conduct a sensitivity analysis by including these variables (see below). Violations of basic assumptions of Cox PH models were also tested, including proportionality of hazards (through Schoenfeld residuals), linearity of effect (through Martingale residuals) and influential observations or survival outliers (through dfbeta residuals). Statistical significance threshold was set to α = 0.05, in light of the non-independence of both exposures (Pearson’s correlation r = 0.76, see [13]) and of the incident events. Associations were deemed significant only if they were significant in the most adjusted model (meaning, Model 3) and showed consistent directions across all the models tested, including sensitivity analyses (see Section 2.5). All the covariates used in the analysis are explained in detail below.

#### Covariates

Data on pre-existing chronic diseases and health conditions were classified as follows. Personal history of cardiovascular disease (CVD: angina, myocardial infarction, revascularization procedures, peripheral artery diseases and cerebrovascular events) was self-reported and confirmed by medical records and therapy in use. Personal history of cancer was self-reported and confirmed by medical records. Participants were considered to have type 2 diabetes (T2D), hyperlipidemia or hypertension if they were taking disease-specific drugs, as assessed by the interviewer. BMI was calculated as weight divided by squared height (kg/m^2^).

Information about sociodemographic factors and lifestyles was obtained at baseline via interviewer-administered questionnaires. Leisure-time physical activity was expressed as daily energy expenditure in metabolic equivalent task hours (MET-h/d) for sport, walking, and gardening [13]. Education was based on the highest qualification attained and was categorized as up to lower secondary (≤8 years of study), upper secondary school (9–13 years of study), and post-secondary education (>13 years of study). Subjects were also classified as never, current, or former smokers (for those who reported not having smoked at all over the previous 12 months or more). Adherence to the traditional Mediterranean Diet (MD) was determined through the MD score developed by Trichopoulou et al. 2003. The MD score ranged from 0 to 9 with increasing values reflecting maximal adherence [26].

#### Sensitivity Analyses

As a sensitivity analysis, the associations with incident events were tested also comparing categorical aging classes as defined below, for each aging clock tested. This allowed for the testing of robustness of the associations against violations of the linearity of effect assumption, which was detected for incident deaths, hospitalizations, as well as fatal/non-fatal CRC, LC, PNC and RNC. To do so, we categorized and compared participants with accelerated Phenotypic Aging (PhenoAge≫CA, namely PhenoAgeAcc ≥ +5 years) and participants with decelerated Phenotypic Aging (PhenoAge CA, PhenoAgeAcc ≤ -5 years), with participant with PhenoAge ≪ CA (namely with PhenoAgeAcc in the interval (-5 years; +5 years), used as a reference class). The same approach was used to compare aging classes for the BloodAgeAcc clock. The number of participants classified in each biological aging category is reported in Table S2a, b.

As a further sensitivity analysis, we extended the analyzed covariates to include lifestyle factors and socioeconomic status. Although these factors are mostly considered ancestral exposures [27], to rule out any potential residual confounding by these factors in the association between biological aging and the incident risk of cancers or related events, we built Cox PH models including smoking, MD adherence score, physical activity, and education as covariates, testing PhenoAgeAcc and BloodAgeAcc both singularly (Model 4) and jointly in fully adjusted regressions (Model 5). Moreover, we carried out interaction analyses of both PhenoAgeAcc and BloodAgeAcc with the analyzed covariates, testing the influence of two-terms interactions for those clocks and covariates showing at least a nominally significant association (p < 0.05) with a given outcome.

As an additional sensitivity analysis, the associations with incident cancer events and related hospitalizations and deaths were tested for each aging clock, excluding events that occurred in the first year of follow-up to rule out potential reverse causality biases.

Similarly, to ensure against violations of the proportional hazards assumption detected in the models testing for incident deaths and hospitalizations, we carried out weighted Cox regressions, a parsimonious alternative of Cox PH regressions which provides well interpretable average effects also in case of non-proportional hazards [28].

The protective associations with PC and BC types underwent further sensitivity analyses to assess their robustness. First, we tested whether the association of BloodAgeAcc with prostate cancer was driven by the protective effect of glucose, in light of the prominent influence of this biomarker on accelerated biological aging and of the reported potential role of glucose and diabetes in lowering prostate-specific antigen levels, which may introduce a detection bias [17]. To do so, we computed the linear regression residuals of BloodAgeAcc vs glucose levels, and tested them for association with incident prostate cancers, as above. Moreover, we tested a potential moderation effect of self-reported familiarity for both prostate and breast cancer, through interaction analysis with BloodAgeAcc. Similarly, we carried out additional interaction analyses for a number of potential risk/protective factors for breast cancer, including reported menopause status at baseline, early menopause (below vs above 45 years of age) and early menarche (below vs above 12 years of age), as well as parity (having no vs one or more children) [29]. Finally, stratified association analyses were carried out for molecular subtypes of breast cancer, based on positivity to estrogen receptor (ER), progesterone receptor (PR) and human epidermal growth factor receptor 2 (HER2), to determine whether the protective association was driven by a specific subtype. These data were obtained via immunohistochemistry, as explained in detail in [30]. ER and PR status were considered positive when more than 1% of tumor cell nuclei were stained, while HER2 status was assessed based on the following IHC scores: a score of 0 or 1+ was interpreted as negative, a score of 3+ as positive, and a score of 2+ required confirmation by fluorescence in situ hybridization (FISH). A positive FISH result confirmed positive HER2 status. If FISH testing was not performed or was absent in the medical records, HER2 status was recorded as a missing value.

### RESULTS

Compared to participants removed from the analysis, the 22,985 analyzed subjects (Figure S1) were significantly younger than the non-analyzed sub-cohort (mean (SD) age 55.6 (11.9) vs 58.3 (13.3) years old), and less frequently women (51.5% vs 58.0%) (Table 1). Similarly, distributions of aging clocks were different, with PhenoAgeAcc and BloodAgeAcc showing slightly lower values compared to non-analyzed subjects. The prevalence of the main chronic health conditions other than cancer was lower in the analyzed cohort, for CVD (5.8% vs 9.1%), T2D (5.0% vs 6.2%), hyperlipidemia (7.9% vs 9.1%) and hypertension (28.7% vs 33.0%), while no significant difference was observed for BMI distribution. This may be explained by the removal of subjects with prevalent cancer history or with lack of blood markers necessary to compute biological aging clocks.

**Table 1:** Baseline characteristics of the population under study.

Variable	Analyzed (N = 22,985)	Not analyzed * (N = 1,340)	Pvalue for difference (p < 0)
**Age (CA, y)**	55.6 (11.9)	58.3 (13.3)	<0.001
**Sex (women, %)**	11,846 (51.5%)	777 (58.0%)	<0.001
**∆PhenoAge (y)**	-9.9 (5.3)	-8.4 (6.8)	<0.001
**∆BloodAge (y)**	-0.9 (7.9)	-2.8 (8.6)	<0.001
**PhenoAgeAcc (y)**	-0.0 (5.2)	0.9 (6.6)	<0.001
**BloodAgeAcc (y)**	-0.1 (6.0)	1.4 (7.1)	<0.001
**Prevalent Health conditions**			
**CVD**	1,307 (5.8%)	120 (9.1%)	<0.001
**T2D**	1,132 (5.0%)	82 (6.2%)	0.05
**Hyperlipidemia**	1,790 (7.9%)	121 (9.1%)	0.10
**Hypertension**	6,547 (28.7%)	440 (33.0%)	<0.001
**BMI (Kg/m^2^)**	28.1 (4.8)	28.2 (5.0)	0.27

We report frequency (%) for categorical variables or, alternatively, mean and standard deviations (SD) for continuous variables. Statistical comparisons of the sub-cohorts were carried out using the Mann-Whitney U test for continuous variables and the Chi-square test for categorical variables, and the resulting p-values were rounded to two decimal places, unless significant. Abbreviations: CVD = cardiovascular disease; T2D= type 2 diabetes; BMI= body mass index; PhenoAgeAcc = ∆PhenoAge regression residuals vs chronological age; BloodAgeAcc = ∆BloodAge regression residuals vs chronological age.

* Subjects with a reported history of cancer at recruitment were excluded, as were those for whom the cancer event could not be ascertained.

The analysis of 865 cancer deaths over 22,985 participants, with a median (interquartile range, IQR) follow-up of 13.1 (2.1) years, revealed significant associations with both aging clocks (Model 3: PhenoAgeAcc Hazard Ratio (HR) [95% confidence interval, CI]: 1.04 [1.03-1.05] per unit increase; BloodAgeAcc: 1.01 [1.00-1.03]), which were stable and concordant across all incremental models tested (Table 2; Table S3a).

Concordantly, in 23,021 with 2,436 incident hospitalizations (follow-up: 12.9 (2.3) years), PhenoAgeAcc showed a significant risk association with incident cancer hospitalizations (Model 3: 1.03 [1.03-1.04]), which was consistent across all models. However, no significant association was observed for BloodAgeAcc when tested alone, and jointly with PhenoAgeAcc (Table 2; Table S3b).

**Figure 2.**
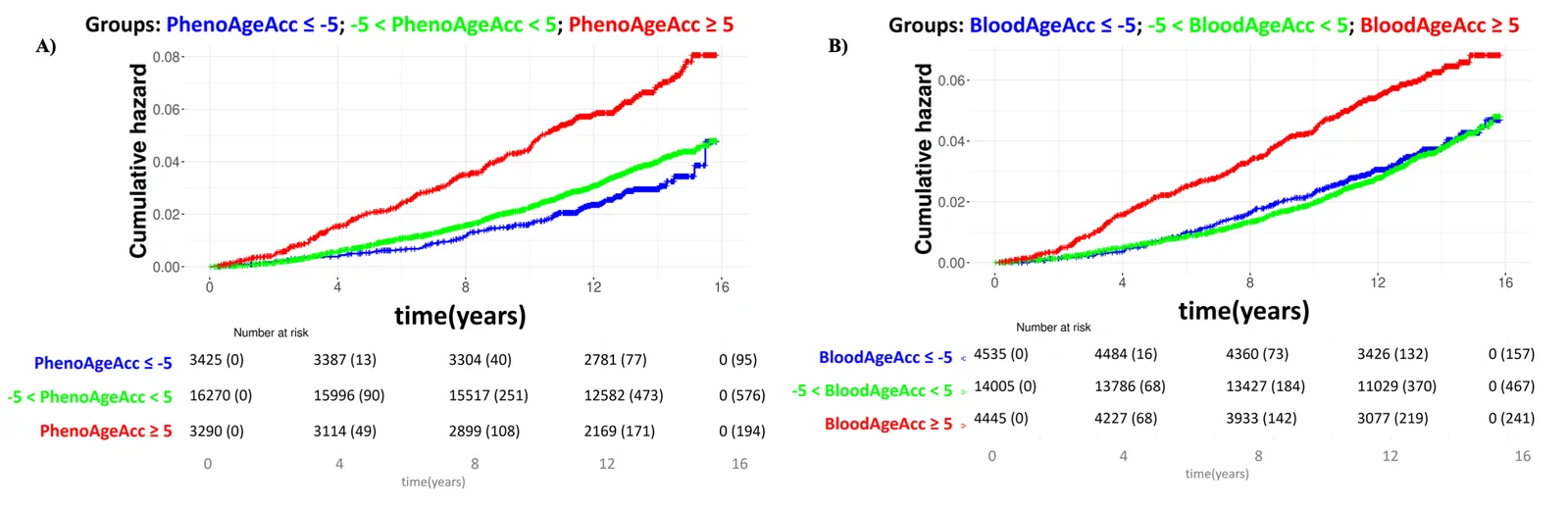
**Cumulative Hazards of incident cancer deaths vs A) PhenoAgeAcc and B) BloodAgeAcc.** Cumulative Hazard Functions (CHF) for cancer deaths are reported for both **A)** PhenoAgeAcc and **B)**BloodAgeAcc categories, along with number of at-risk subjects and number of events within each class during follow-up. Legend: green = -5 y < PhenoAgeAcc/BloodAgeAcc < +5 y; red = PhenoAgeAcc/BloodAgeAcc ≥ +5 y; blue = PhenoAgeAcc/BloodAgeAcc ≤ -5 y.

**Figure 3.**
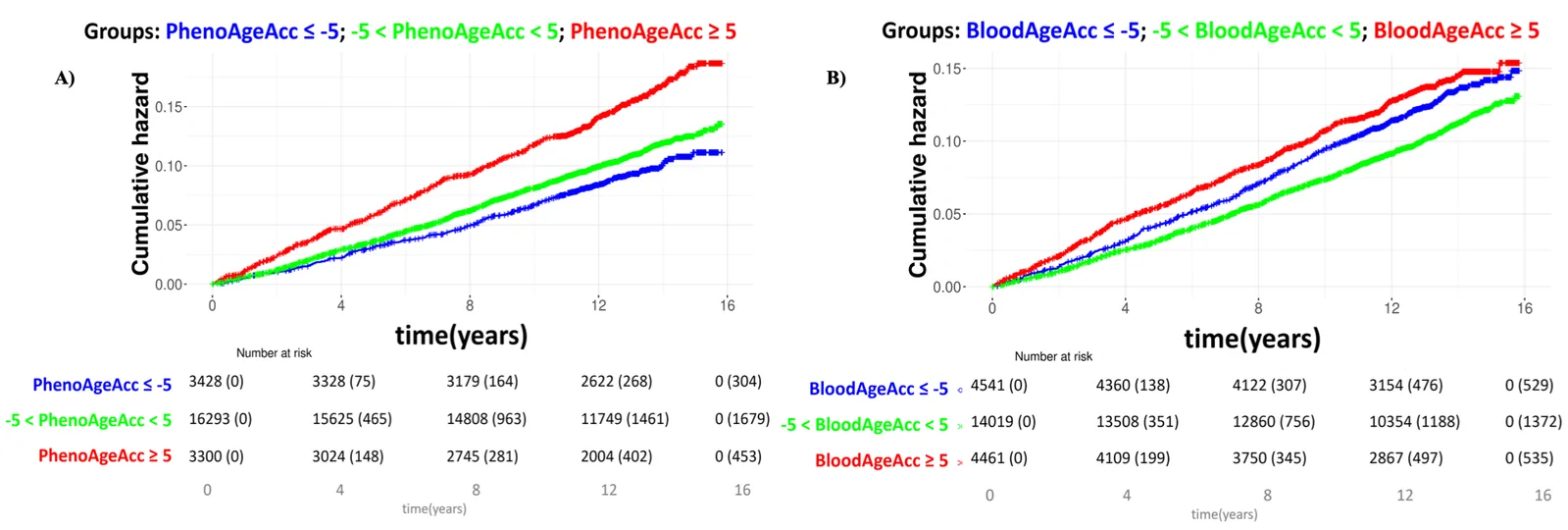
**Cumulative Hazards of incident first cancer hospitalizations vs A) PhenoAgeAcc and B) BloodAgeAcc.** Cumulative Hazard Functions (CHF) for incident first cancer hospitalizations are reported for both **A)** PhenoAgeAcc and **B)**BloodAgeAcc categories, along with number of at-risk subjects and number of events within each class during follow-up. Legend: green = -5 y < PhenoAgeAcc/BloodAgeAcc < +5 y; red = PhenoAgeAcc/BloodAgeAcc ≥ +5 y; blue = PhenoAgeAcc/BloodAgeAcc ≤ -5 y.

These trends were confirmed when we compared subjects belonging to the different biological aging categories (Table S4a, b). Indeed, participants with notably accelerated Phenotypic Aging (i.e. with PhenoAgeAcc ≥ 5 years) showed a steep increase in the cumulative hazard function of cancer deaths, compared to participants with PhenoAge⁓CA (-5 years < PhenoAgeAcc < +5 years), while participants with notably decelerated Phenotypic Aging (with PhenoAgeAcc ≤ -5 years) showed a notable reduction of risk (Fig. 2A). Similarly, participants biologically older than their CA in terms of BloodAge (i.e. with BloodAgeAcc ≥ 5 years) showed a significant increase in the cumulative hazard function of cancer deaths (Fig. 2B), compared to participants with BloodAge ⁓ CA (-5 y < BloodAgeAcc < +5 y), while biologically younger participants (with BloodAgeAcc ≤ -5 years) showed an opposite but non-significant trend (Table S4a). Similar trends of associations were observed with the incident risk of hospitalizations for PhenoAgeAcc (Figure 3a) but not for BloodAgeAcc classes (Fig. 3B, Table S4b). Similarly, a weighted Cox PH regression analysis to ensure robustness against violation of the linearity assumption confirmed risk associations with both incident deaths and hospitalizations for both aging clocks (Table S5a, b).

An analysis of the incident risk of single cancer types – including both fatal and non-fatal events (Table S6a-f) – revealed a significant association of PhenoAgeAcc with LC over 208 incident cases and 22,878 participants, being each year increase associated with a 4.6 (2.2-7.0)% increase of incident risk. A similar association was observed with RNC, over 94 incident cases and 22,895 participants, with a 5.2 (1.6-8.9)% increased risk for yearly increase in PhenoAgeAcc.

**Table 2:** Associations between aging clocks tested and incident cancer-related risks.

Aging Clock	MODEL 1 - PhenoAge HR [95% CI] (*p* value)	MODEL 2 – PhenoAge HR [95% CI] (*p* value)	MODEL 1 - BloodAge HR [95% CI] (*p* value)	MODEL 2 – BloodAge HR [95% CI] (*p* value)	MODEL 3 HR [95% CI] (*p* value)	Cancer Event (nr)
**PhenoAgeAcc**	**1.047 [1.037-1.058] (p<0.001)**	**1.045 [1.033-1.057] (p<0.001)**	.	.	**1.042 [1.030-1.054] (p<0.001)**	**Death (865)**
**BloodAgeAcc**	.	.	**1.025 [1.015-1.036]** (p<0.001)	**1.021 [1.010-1.032]** ( p<0.001)	**1.014 [1.003-1.025] (p=0.015)**	
**PhenoAgeAcc**	**1.031 [1.024-1.038] (p<0.001)**	**1.033 [1.025-1.041] (p<0.001)**	.	.	**1.034 [1.026-1.042] (p<0.001)**	**First Hospitalization (2,436)**
BloodAgeAcc	.	.	1.004 [1.000-1.011] (p=0.19)	1.002 [0.995-1.008] (p=0.61)	0.996 [0.989-1.003] (p=0.28)	
PhenoAgeAcc	1.013 [0.991-1.035] (p=0.24)	1.003 [0.979-1.028] (p=0.80)	.	.	1.007 [0.982-1.032] (p=0.60)	**BC (314)**
**BloodAgeAcc**	.	.	0.985 [0.967-1.004] (p=0.13)	**0.979 [0.959-0.999] (p=0.038)**	**0.978 [0.958-0.998] (p=0.034)**	
PhenoAgeAcc	1.015 [0.996-1.033] (p=0.12)	1.017 [0.996-1.038] (p=0.11)	.	.	1.019 [0.998-1.040] (p=0.08)	CRC (380)
BloodAgeAcc	.	.	0.995 [0.980-1.011] (p=0.57)	0.995 [0.978-1.012] (p=0.53)	0.992 [0.975-1.009] (p=0.34)	
**PhenoAgeAcc**	**1.050 [1.029-1.071] (p<0.001)**	**1.046 [1.023-1.069] (p<0.001)**	.	.	**1.046 [1.022-1.070] ( p<0.001)**	**LC (208)**
BloodAgeAcc	.	.	1.017 [0.996-1.039] (p=0.12)	1.011 [0.988-1.034] (p=0.36)	1.002 [0.980-1.026] (p=0.84)	
PhenoAgeAcc	0.984 [0.965-1.003] (p=0.10)	0.990 [0.969-1.010] (p=0.33)	.	.	0.995 [0.973-1.016] (p=0.61)	**PC (387)**
**BloodAgeAcc**	.	.	**0.980 [0.964-0.995] (p=0.009)**	**0.982 [0.966-0.998] (p=0.030)**	**0.983 [0.967-1.000] (p=0.046)**	
**PhenoAgeAcc**	**1.040 [1.005-1.076] (p=0.025)**	**1.039 [1.002-1.078] (p=0.040)**	.	.	1.030 [0.991-1.071] (p=0.14)	**PNC (86)**
**BloodAgeAcc**	.	.	**1.046 [1.011-1.081] (p=0.009)**	**1.047 [1.011-1.084] (p=0.009)**	**1.042 [1.005-1.079] (p=0.024)**	
**PhenoAgeAcc**	**1.053 [1.023-1.084] (p<0.001)**	**1.052 [1.018-1.089] (p=0.003)**	.	.	**1.052 [1.016-1.089] (p=0.004)**	**RNC (94)**
BloodAgeAcc	.	.	1.032 [0.999-1.066] (p=0.06)	1.013 [0.979-1.048] (p=0.47)	1.003 [0.969-1.039] (p=0.85)	

Risk estimates for (a) cancer deaths, (b) first cancer hospitalizations and (c) specific fatal/nonfatal (breast, colorectal, lung, prostate, pancreatic and renal) cancer risks are expressed as Hazard Ratios (HRs) with 95% Confidence Intervals (CIs) for annual increase in PhenoAgeAcc/BloodAgeAcc, as calculated through Cox PH incrementally adjusted multivariable models. P values are rounded to two decimal places unless significant. Significant associations (p<0.05) are highlighted in bold. Associations of all the covariates included in the models are reported in the Supplementary Results. Legend: Model 1-PhenoAge: age + sex + PhenoAgeAcc; Model 2-PhenoAge: Model 1 + prevalent health conditions (CVD, T2D, hyperlipidemia, hypertension, BMI); Model 1-BloodAge: age + sex + BloodAgeAcc; Model 2-BloodAge: Model 1 + prevalent health conditions (CVD, T2D, hyperlipidemia, hypertension, BMI); Model 3: age + sex + prevalent health conditions (CVD, T2D, hyperlipidemia, hypertension, BMI + PhenoAgeAcc + BloodAgeAcc.

Abbreviations: CVD: cardiovascular disease; T2D: type 2 diabetes; BMI: body mass index; BC: breast cancer; CRC: colorectal cancer; LC: lung cancer; PC: prostate cancer, PNC: pancreatic cancer; RNC: renal cancer; PhenoAgeAcc = ∆PhenoAge regression residuals vs chronological age; BloodAgeAcc= ∆BloodAge regression residuals vs chronological age.

Conversely, BloodAgeAcc showed weaker but protective associations, with both incident BC (314 incident cases out of 11,772 participants: 2.2 (0.2-4.2)% reduction of risk) and incident PC (387 incident cases over 11,087 participants: 1.70 (0.03-3.3)% reduction of risk). Additionally, BloodAgeAcc also revealed a significant risk association with incident PNC (over 86 incident cases and 22,891 participants: 4.2 (0.5-7.9)% increase of risk per year increase of BloodAgeAcc). Again, these associations were confirmed in sensitivity analyses testing incident cancer risks for specific causes vs biological aging categories, to overcome violations of the proportional hazard’s assumption, where applicable (i.e. for all fatal/non-fatal events except breast and prostate cancer) (Table S7a-d).

These associations were substantially confirmed in sensitivity analyses that tested all risks of incident cancer by specific causes, cancer-related deaths, and hospitalizations vs BloodAgeAcc and PhenoAgeAcc, excluding events that occurred during the first year of follow-up (Table S8).

Similarly, the inclusion of lifestyle and socioeconomic covariates did not substantially change the observed associations between the biological aging clocks (PhenoAgeAcc, BloodAgeAcc) and the incident events of interest, although some attenuation was observed for the association of BloodAgeAcc with prostate cancer, when tested jointly with PhenoAgeAcc (Table S9). This further supports the robustness of our observations and suggests that the estimated effects were not heavily confounded by these factors. An interaction analysis of the aging clocks tested and covariates revealed two significant associations with incident cancer hospitalization risk: one for the PhenoAgeAcc-by-(upper secondary) education interaction, which showed a significant negative marginal association (0.975 [0.959-0.993]); and one for the PhenoAgeAcc-by-smoking interaction, which showed a marginally significant positive marginal association (1.020 [1.001-1.038]; Table S10a-f).

Further sensitivity analyses on the link between BloodAgeAcc and PC revealed that the protective association observed was independent on glucose levels (Table S11), but did not highlight any statistically significant influence of the interaction term between PC familiarity and BloodAgeAcc (Table S12). Similarly, no statistically significant moderations were observed for several risk/protective factors on breast cancer risk, including menopause, early menopause or menarche and parity (having no vs one or more children), as well as familiarity, although for the latter the interaction effect approached statistical significance (Table S13a-e). A further analysis stratified by BC familiarity revealed that those reporting no family history of breast cancer were more protected - by 2.6(0.4-4.7)% per year increase of BloodAgeAcc - against incident BC risk, compared to those who reported familiarity, where the association did not show any evident trend (Table S14a, b).

A stratified analysis by molecular subtypes of BC revealed no substantial discrepancy in the direction of association between positive (ER+, PR+ and HER2+) versus negative cases, although these did not always reach statistical significance due to the further reduction in sample size of the stratified analyses (Table S15a-f).

## Discussion

We analysed different blood-based markers of biological aging to test their power to predict clinical risks related to cancer – namely deaths and hospitalizations – as well as the incident risk of fatal/non-fatal events for different cancer types, including breast, prostate, colorectal, lung, pancreatic and renal cancer.

We observed statistically significant associations with increased cancer mortality for both PhenoAgeAcc and BloodAgeAcc, which is in line with previous evidence from large independent cohorts: indeed, PhenoAge acceleration was associated with an increased all-cause and CVD mortality in cancer survivors from the NHANES cohort [31], and several domains of biological aging based on blood immune, renal and hepatic markers showed a significant association with cancer mortality in the UK Biobank [32]. Similar evidence was also observed for site-specific cancer mortality – including oral, esophageal, gastric, liver, pancreatic, lung and kidney cancer, as well as multiple myeloma, and leukemia [33, 34]. Similarly, risk associations were also reported between total cancer mortality and DNA methylation (DNAm) PhenoAge acceleration, which was based on surrogate epigenetic markers of PhenoAge [33, 25, 34], as well as with other DNAm age accelerations [15]. The influences of PhenoAgeAcc and BloodAgeAcc were independent of each other, which aligns with previous evidence of independent effects on all-cause mortality for these two clocks in the Moli-sani cohort [13]. Similarly DNAm clocks showed predictions of cancer incidence and mortality, independent of other biological aging estimators like telomere attrition [15]. The analysis of incident cancer hospitalizations also revealed a statistically significant increase of risk as biological aging acceleration increased, but only for PhenoAgeAcc. To our knowledge, no targeted association analyses between biological aging markers and cancer hospitalizations in a population cohort have been published.

The analysis of incident fatal/non-fatal cancer cases for different body sites revealed both increased and decreased cancer risks associated with biological aging acceleration: BloodAgeAcc was indeed associated with an increased risk of PNC, while PhenoAgeAcc showed an increased risk of LC and RNC. Risk associations for these cancers have been reported elsewhere, both for epigenetic clocks [35–37] and for blood-based markers like PhenoAge acceleration and the Klemera-Doubal clock [33, 17].

Interestingly, a weak protective association of BloodAgeAcc was found with both BC and PC. This is partly concordant with the protective influence of PhenoAge acceleration on incident prostate cancer previously reported in the UK Biobank [33, 17], and in agreement with mendelian randomization evidence suggesting a protective effect of the (DNAm) GrimAge acceleration against PC [19]. Similarly, another DNAm aging (MRscore) acceleration showed a protective influence against incident breast cancer risk in a longitudinal German population cohort [35], concordant with our findings. This is in contrast with the increased risk of BC reported for accelerated epigenetic aging in prospective cohorts [33, 17], case-control [38] and case-cohort analyses [18]. Among other prior contradictory findings is the positive association of age-adjusted PhenoAge acceleration with BC and CRC [17], which we did not find here. Different aspects may explain these contrasting findings. First, most of these clocks are based on substantially different algorithms and markers, are weakly correlated with each other and may tag different aspects of biological aging [17]. Alternatively, biases that often influence observational research, such as reverse causality (e.g. cancer influencing the epigenome) and residual confounding (e.g. unmeasured confounders or imprecisely measured association between tested aging clocks and cancer [39]) may play a role. Finally, differential outcomes and adjustment of survival models may also help explain these discrepancies. Nonetheless, our findings were quite robust against potential confounding by lifestyles and socioeconomic status. Of note, interaction analyses revealed interesting evidence suggesting that in participants with an upper secondary education the positive influence of PhenoAge acceleration on incident cancer hospitalization risk was somehow attenuated (by 2.5(0.7-4.1)% per year increase in PhenoAgeAcc) compared to those with primary or lower education, while it was slightly increased (by 2.0(0.1-3.8)%) in smokers compared to non-smokers. Since this evidence is quite weak and has never been reported before to our knowledge, it warrants further replication studies. Similarly, the partly discrepant findings with previous studies suggest analyses in independent longitudinal cohorts, with larger follow-up time, as well as powerful mendelian randomization analyses to clarify the causality link between pure blood-based biological aging estimators and different cancer risks, as done in the past for DNAm ages [19]. While it would be tempting to see the immediate translational impact of our findings, such studies are fundamental before moving to translating scientific evidence into public health and prevention actions.

## Strengths and limitations

Our study presents different points of strengths. Firstly, it is a large prospective cohort study, with long follow-up, implying sufficient statistical power. Secondly, the linkage with medical records conferred us the potential to identify diverse incident cancer events in several body sites, along with related clinical risks like deaths and hospitalizations. Thirdly, due to the availability of a wide range of blood biomarkers, we were able to estimate and test two different systemic biological aging measures, which have been previously reported to predict independent portions of risk in all-cause mortality [13]. This evidence was further supported in our study by cancer mortality. The use of two different types of biological aging indices represents an important novelty, along with the wealth of outcomes analyzed, which allowed us to untangle the relationship between systemic biological aging and cancer risks in an unprecedentedly comprehensive way. Fourthly, we carried out several sensitivity analyses to assess the robustness of the associations detected. Lastly, our study represents one of the first replication studies for findings reported on PhenoAge acceleration in other large prospective cohorts, prominently the UK Biobank [33, 17], which were previously warranted [40].

However, our study also includes some limitations. Our PhenoAge measure could not be computed as described in the original manuscript [25] due to the lack of ALP, but a proxy highly correlated measure with comparable performance [13] was used instead. Additionally, our sensitivity analyses used to explore how specific cancer subtypes are influenced by biological aging are limited, due to the lack of molecular characterization data for most of the patients followed-up. Similarly, as these findings were made in a population from a small region in central Italy, their generalizability is limited, despite evidence of concordant findings in independent European cohorts (e.g. UK Biobank; [17]). A selection bias may also be present due to the unavoidable removal of subjects with cancer history or missing blood markers info, which led to the inclusion of younger subjects in the analysis. Moreover, residual and unmeasured confounding cannot be ruled out in observational studies like ours, which warrants further causal inference studies like Mendelian randomization to clarify the causality link between biological aging and cancer. Finally, the availability of other systemic biological aging (e.g. epigenetic) clocks would have allowed us to make our study directly comparable with several other studies in the field, but these were available only for a minority of the samples (N~2,000), which makes it unfeasible to test the joint influence of these clocks with BloodAge and PhenoAge accelerations, given the rarity of cancer events. We are currently working towards increasing our sample number with epigenomic data, so as to reach a sufficient statistical power and thus carry out these analyses in the future. Nonetheless, it should be noticed that, although DNAm aging clocks previously showed associations with both cancer incidence and mortality [12, 18], these aging markers are unlikely to be routinely used in large populations since they are based on expensive and time-consuming techniques. On the contrary, blood-based biological aging markers may represent cost-effective tools for screening the risk of cancer and related clinical risks in aging population settings.

## Supplementary Materials

The Supplementary data can be found online at: www.aginganddisease.org/EN/10.14336/AD.2025.0204.



## Data Availability

The data underlying this article will be shared upon reasonable request to the corresponding author. The data are stored in an institutional repository (https://repository.neuromed.it) and access is restricted by the ethics approval and the legislation of the European Union.
