# Supplementary material for: Exploring the Complex Relationship between Biological Aging and Cancer in a Prospective Italian Population Cohort
Source: Aging Dis. 2025 May 27;17(3):1710–22. doi: 10.14336/AD.2025.0204 (PMC13061564; doi:10.14336/AD.2025.0204)
Supplement: Supplementary file 1 [file AD-17-3-1710-s.pdf]

Exploring the Complex Relationship between Biological Aging and Cancer in a Prospective Italian Population Cohort

Martina Morelli, Antonietta Pepe, Simona Costanzo, Teresa Panzera, Sara Magnacca, Amalia De Curtis, Maria Loreto Muñoz Venegas, Chiara Cerletti, Maria Benedetta Donati, Giovanni de Gaetano, Licia Iacoviello, Alessandro Gialluisi, on behalf of the Moli-sani Study Investigators

Supplementary Results

Figure S1. Flowchart of the study participants.

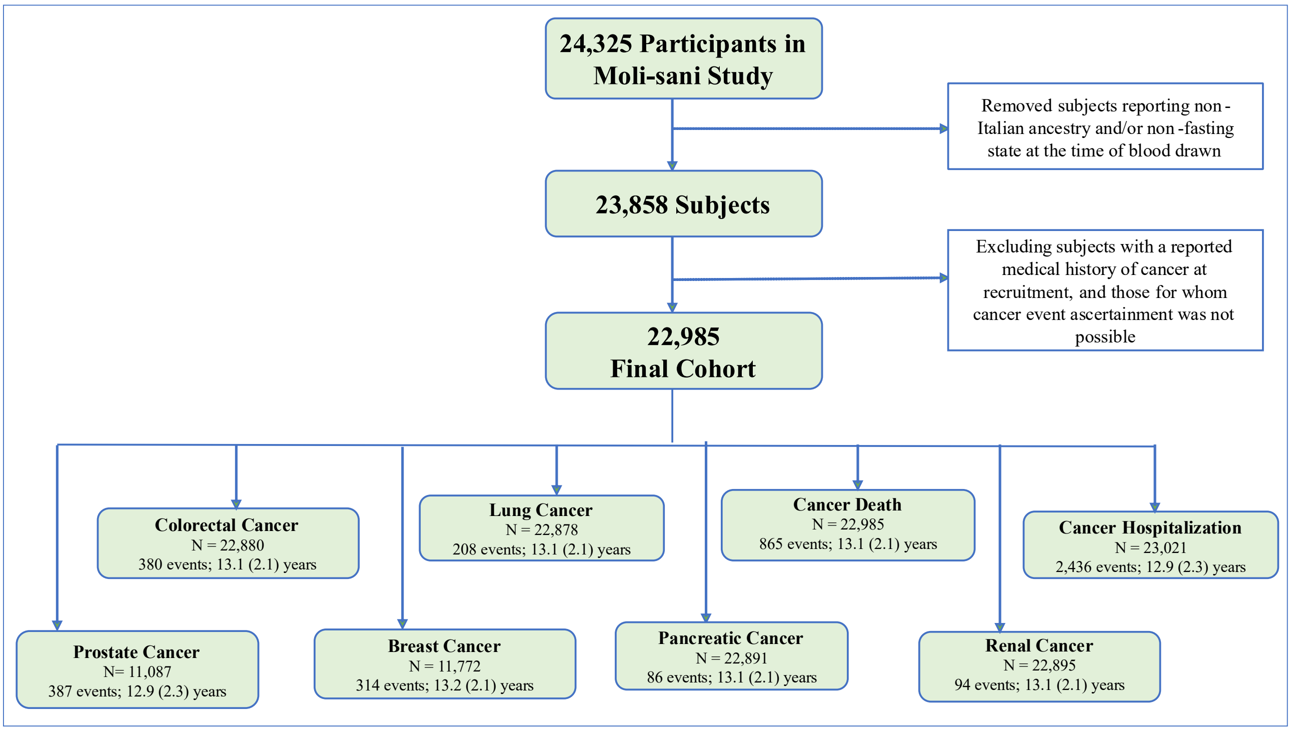

Actual sample size, number of events and median (interquartile range) follow-up time are reported for each analyzed outcome.

Table S1. Missing rate for **a)** outcome and **b)** covariates measures.

a)

Variable	Observations	# Missing	Missing (%)
Sex	24325	0	0.00
Age	24325	0	0.00
CVD	24023	302	1.24
T2D	24017	308	1.27
Hyperlipidemia	24092	233	0.96
Hypertension	24149	176	0.72
BMI	24308	17	0.07
Education	24286	39	0.16
Physical activity	24325	0	0.00
MD	24221	104	0.43
Smoking	24296	29	0.12

b)

Cancer Event	Observations	# Missing	Missing (%)
Death	24285	40	0.16
First Hospitalization	24325	0	0.00
BC	12061	562	2.31
CRC	23362	963	3.96
LC	23362	963	3.96
PC	11283	419	1.72
PNC	23376	949	3.89
RNC	23380	945	3.89

Abbreviations: CVD: cardiovascular disease; T2D: type 2 diabetes; BMI: body mass index; MD: Mediterranean diet score; BC: breast cancer; CRC: colorectal cancer; LC: lung cancer; PC: prostate cancer, PNC: pancreatic cancer; RNC: renal cancer.

Table S2. Frequency of biological aging categories for **a)** PhenoAgeAcc and **b)** BloodAgeAcc.

a)

**Aging category**	**Counts**	**Frequency (%)**	**Cumulative frequency (%)**
-5 y < PhenoAgeAcc < +5 y **(reference)**	16833	70.6	70.6
PhenoAgeAcc ≥ 5 years **(accelerated aging class)**	3475	14.6	85.1
PhenoAgeAcc ≤ -5 years **(decelerated aging class)**	3550	14.9	100.0

b)

**Aging category**	**Counts**	**Frequency (%)**	**Cumulative frequency (%)**
-5 y < BloodAgeAcc < +5 y **(reference)**	14420	60.4	60.4
BloodAgeAcc ≥ 5 years **(accelerated aging class)**	4730	19.8	80.3
BloodAgeAcc ≤ -5 years **(decelerated aging class)**	4708	19.7	100.0

These classifications were based on PhenoAge and BloodAge acceleration indices (i.e. regression residuals of each Δage vs chronological age (CA) of individuals; N = 23,858). PhenoAgeAcc: ΔPhenoAge regression residuals vs chronological age; BloodAgeAcc: ΔBloodAge regression residuals vs chronological age.

Table S3. Associations of aging clocks with cancer **a)** death and **b)** first hospitalization risk.

a)

Variable	MODEL 1 - PhenoAge<br>HR [95% CI]<br>(*p* value)	MODEL 2 – PhenoAge<br>HR [95% CI]<br>(*p* value)	MODEL 1 - BloodAge<br>HR [95% CI]<br>(*p* value)	MODEL 2 – BloodAge<br>HR [95% CI]<br>(*p* value)	MODEL 3<br>HR [95% CI]<br>(*p* value)
Sex (men)	**1.885 [1.636-2.173] (p<0.001)**	**1.911 [1.647-2.217] (p<0.001)**	**2.071 [1.800-2.383] (p<0.001)**	**2.079 [1.795-2.407] (p<0.001)**	**1.913 [1.649-2.220] (p<0.001)**
Age	**1.087 [1.080-1.093] (p<0.001)**	**1.088 [1.081-1.096] (p<0.001)**	**1.086 [1.079-1.092] (p<0.001)**	**1.087 [1.079-1.094] (p<0.001)**	**1.088 [1.081-1.096] (p<0.001)**
CVD	.	0.976 [0.767-1.243] (p=0.84)	.	1.000 [0.785-1.274] (p=1.00)	0.949 [0.744-1.210] (p=0.67)
T2D	.	1.206 [0.958-1.518] (p=0.11)	.	**1.395 [1.114-1.746] (p=0.004)**	1.195 [0.949-1.504] (p=0.13)
Hyperlipidemia	.	0.999 [0.796-1.253] (p=0.99)	.	0.962 [0.766-1.207] (p=0.74)	1.001 [0.798-1.257] (p=0.99)
Hypertension	.	**0.847 [0.726-0.989] (p=0.036)**	.	**0.849 [0.727-0.991] (p=0.038)**	**0.829 [0.710-0.970] (p=0.018)**
BMI	.	1.016 [1.000-1.031] (p=0.05)	.	**1.024 [1.008-1.040] (p=0.002)**	1.015 [0.999-1.030] (p=0.07)
PhenoAgeAcc	**1.047 [1.037-1.058] (p<0.001)**	**1.045 [1.033-1.057] (p<0.001)**	.	.	**1.042 [1.029-1.054] (p<0.001)**
BloodAgeAcc	.	.	**1.025 [1.015-1.036] (p<0.001)**	**1.021 [1.010-1.032] (p<0.001)**	**1.014 [1.003-1.025] (p=0.015)**

b)

Variable	MODEL 1 - PhenoAge<br>HR [95% CI]<br>(*p* value)	MODEL 2 – PhenoAge<br>HR [95% CI]<br>(*p* value)	MODEL 1 - BloodAge<br>HR [95% CI]<br>(*p* value)	MODEL 2 – BloodAge<br>HR [95% CI]<br>(*p* value)	MODEL 3<br>HR [95% CI]<br>(*p* value)
Sex (men)	**1.454 [1.340-1.577] (p<0.001)**	**1.460 [1.342-1.589] (p<0.001)**	**1.538 [1.419-1.667] (p<0.001)**	**1.542 [1.419-1.676] p<0.001)**	**1.460 [1.342-1.588] (p<0.001)**
Age	**1.047 [1.044-1.051] (p<0.001)**	**1.049 [1.045-1.053] (p<0.001)**	**1.047 [1.043-1.050] (p<0.001)**	**1.047 [1.043-1.051] (p<0.001)**	**1.049 [1.045-1.053] (p<0.001)**
CVD	.	0.844 [0.713-1.000*] (p=0.05)	.	0.889 [0.750-1.052] (p=0.17)	0.851 [0.719-1.009] (p=0.06)
T2D	.	1.084 [0.924-1.272] (p=0.32)	.	**1.236 [1.057-1.445] (p=0.008)**	1.088 [0.927-1.276] (p=0.30)

Hyperlipidemia	.	1.080 [0.936-1.246] (p=0.29)	.	1.050 [0.910-1.211] (p=0.51)	1.079 [0.935-1.245] (p=0.30)
Hypertension	.	0.956 [0.869-1.053] (p=0.36)	.	0.975 [0.885-1.074] (p=0.60)	0.962 [0.873-1.060] (p=0.44)
BMI	.	1.002 [0.992-1.011] (p=0.73)	.	**1.010 [1.000-1.019] (p=0.039)**	1.002 [0.993-1.011] (p=0.69)
PhenoAgeAcc	**1.031 [1.024-1.038] (p<0.001)**	**1.033 [1.025-1.041] (p<0.001)**	.	.	**1.034 [1.026-1.042] (p<0.001)**
BloodAgeAcc	.	.	1.004 [0.998-1.011] (p=0.19)	1.002 [0.995-1.009] (p=0.61)	0.996 [0.989-1.003] (p=0.28)

Hazard Ratios (HRs) with 95% Confidence Intervals (CIs) for **a)** cancer deaths and **b)** first cancer hospitalizations are reported for all the covariates included in incrementally adjusted Cox PH models.
Legend: Model 1-PhenoAge: age + sex + PhenoAgeAcc; Model 2-PhenoAge: Model 1 + prevalent health conditions (CVD, T2D, hyperlipidemia, hypertension, BMI); Model 1-BloodAge: age + sex + BloodAgeAcc; Model 2-BloodAge: Model 1 + prevalent health conditions (CVD, T2D, hyperlipidemia, hypertension, BMI); Model 3: age + sex + prevalent health conditions (CVD, T2D, hyperlipidemia, hypertension, BMI) + PhenoAgeAcc + BloodAgeAcc. P values are rounded to two decimal places unless significant.
Abbreviations: CVD: cardiovascular disease; T2D: type 2 diabetes; BMI: body mass index; PhenoAgeAcc: ΔPhenoAge regression residuals vs chronological age; BloodAgeAcc: ΔBloodAge regression residuals vs chronological age.
***** Model 3: the actual superior limit of the HR confidence interval is 0.9995.

Table S4. Associations between aging categories and cancer **a)** death and **b)** first hospitalization risk.

a)

Variable	HR	HRinf	HRsup	P value
Sex (men)	**1.96**	**1.69**	**2.28**	**<0.001**
Age	**1.09**	**1.08**	**1.09**	**<0.001**
CVD	0.97	0.76	1.23	0.79
T2D	**1.26**	**1.00**	**1.58**	**0.047**
Hyperlipidemia	0.99	0.79	1.24	0.92
Hypertension	**0.84**	**0.72**	**0.98**	**0.024**
BMI	**1.02**	**1.00**	**1.03**	**0.032**
PhenoAgeAcc ≥ 5 y	**1.53**	**1.28**	**1.82**	**<0.001**
PhenoAgeAcc ≤ -5 y	0.81	0.64	1.01	0.07
BloodAgeAcc ≥ 5 y	**1.19**	**1.01**	**1.41**	**0.037**
BloodAgeAcc ≤ -5 y	0.89	0.74	1.07	0.20

b)

Variable	HR	HRinf	HRsup	P value
Sex (men)	**1.96**	**1.36**	**1.61**	**<0.001**
Age	**1.09**	**1.04**	**1.05**	**<0.001**
CVD	0.97	0.72	1.02	0.08
T2D	1.26	0.97	1.33	0.12
Hyperlipidemia	0.99	0.93	1.24	0.34
Hypertension	0.84	0.87	1.06	0.44
BMI	1.02	0.99	1.01	0.41
PhenoAgeAcc ≥ 5 y	**1.53**	**1.23**	**1.53**	**<0.001**
PhenoAgeAcc ≤ -5 y	**0.81**	**0.73**	**0.94**	**0.004**
BloodAgeAcc ≥ 5 y	1.19	0.94	1.16	0.46
BloodAgeAcc ≤ -5 y	0.89	0.95	1.17	0.34

Associations of the different biological aging categories are reported compated to the relevant reference classes (-5 y < BloodAgeAcc < +5 y and -5 y < PhenoAgeAcc < +5 y, respectively) are reported for the most enriched model (Model 3: age + sex + prevalent health conditions (CVD, T2D, hyperlipidemia, hypertension, BMI) + PhenoAgeAcc + BloodAgeAcc. P values are rounded to two decimal places unless significant. Abbreviations: CVD: cardiovascular disease; T2D: type 2 diabetes; BMI: body mass index; HR: Hazard Ratio; HRinf/HRsup: Lower and Upper limit of the HR confidence interval; PhenoAgeAcc: ΔPhenoAge regression residuals vs chronological age; BloodAgeAcc: ΔBloodAge regression residuals vs chronological age.

Table S5. Weighted Cox Regressions Models of incident **a)** cancer death and **b)** first cancer hospitalization risk.

a)

**Variable**	**HR**	**HRinf**	**HRsup**	**P value**
Sex (men)	**1.83**	**1.46**	**2.30**	**<0.001**
Age	**1.09**	**1.08**	**1.10**	**<0.001**
CVD	0.97	0.71	1.33	0.86
T2D	1.19	0.91	1.54	0.20
Hyperlipidemia	0.99	0.72	1.37	0.95
Hypertension	**0.78**	**0.61**	**0.99**	**0.042**
BMI	1.00	0.98	1.02	0.83
PhenoAgeAcc	**1.04**	**1.02**	**1.06**	**<0.001**
BloodAgeAcc	**1.01**	**1.00**	**1.03**	**0.034**

b)

**Variable**	**HR**	**HRinf**	**HRsup**	**P value**
Sex (men)	**1.42**	**1.27**	**1.58**	**<0.001**
Age	**1.05**	**1.05**	**1.05**	**<0.001**
CVD	0.85	0.69	1.05	0.14
T2D	1.06	0.88	1.27	0.55
Hyperlipidemia	1.04	0.86	1.25	0.71
Hypertension	0.95	0.83	1.08	0.43
BMI	1.01	0.99	1.02	0.25
PhenoAgeAcc	**1.03**	**1.02**	**1.04**	**<0.001**
BloodAgeAcc	1.00	0.99	1.01	0.50

Hazard Ratios (HRs) with 95% Confidence Intervals (CIs) and relevant p-values for **a)** cancer deaths and **b)** first cancer hospitalizations are reported for all the covariates included in incrementally adjusted Cox PH models. P values are rounded to two decimal places unless significant. Abbreviations: CVD: cardiovascular disease; T2D: type 2 diabetes; BMI: body mass index; HR: Hazard Ratio; HRinf/HRsup: Lower and Upper limit of the HR confidence interval; PhenoAgeAcc: ΔPhenoAge regression residuals vs chronological age; BloodAgeAcc: ΔBloodAge regression residuals vs chronological age.

Table S6. Associations of aging clocks with incident fatal/nonfatal **a)** breast, **b)** colorectal, **c)** lung, **d)** prostate, **e)** pancreatic and **f)** renal cancers risks.

a)

Variable	MODEL 1 - PhenoAge HR [95% CI] (*p* value)	MODEL 2 – PhenoAge HR [95% CI] (*p* value)	MODEL 1 - BloodAge HR [95% CI] (*p* value)	MODEL 2 – BloodAge HR [95% CI] (*p* value)	MODEL 3 HR [95% CI] (*p* value)
Sex (men)	.	.	.	.	.
Age	**1.013 [1.004-1.023] (p=0.006)**	1.010 [0.998-1.021] (p=0.11)	**1.013 [1.003-1.022] (p=0.009)**	1.008 [0.996-1.019] (p=0.19)	1.008 [0.997-1.020] (p=0.17)
CVD	.	1.215 [0.692-2.132] (p=0.50)	.	1.248 [0.712-2.189] (p=0.44)	1.240 [0.706-2.175] (p=0.45)
T2D	.	0.985 [0.534-1.815] (p=0.96)	.	1.037 [0.568-1.893] (p=0.91)	1.007 [0.546-1.857] (p=0.98)
Hyperlipidemia	.	1.151 [0.752-1.760] (p=0.52)	.	1.133 [0.741-1.732] (p=0.56)	1.140 [0.745-1.744] (p=0.55)
Hypertension	.	0.937 [0.700-1.254] (p=0.66)	.	0.977 [0.729-1.310] (p=0.88)	0.977 [0.728-1.310] (p=0.88)
BMI	.	1.021 [0.997-1.045] (p=0.08)	.	**1.027 [1.004-1.050] (p=0.020)**	**1.025 [1.001-1.050] (p=0.040)**
PhenoAgeAcc	1.013 [0.991-1.035] (p=0.24)	1.003 [0.979-1.028] (p=0.80)	.	.	1.007 [0.982-1.032] (p=0.60)
BloodAgeAcc	.	.	0.985 [0.967-1.004] (p=0.13)	**0.979 [0.959-0.999] (p=0.038)**	**0.978 [0.958-0.998] (p=0.034)**

b)

Variable	MODEL 1 - PhenoAge HR [95% CI] (*p* value)	MODEL 2 – PhenoAge HR [95% CI] (*p* value)	MODEL 1 - BloodAge HR [95% CI] (*p* value)	MODEL 2 – BloodAge HR [95% CI] (*p* value)	MODEL 3 HR [95% CI] (*p* value)
Sex (men)	**1.496 [1.216-1.841] (p<0.001)**	**1.574 [1.265-1.959] (p<0.001)**	**1.542 [1.257-1.892] (p<0.001)**	**1.625 [1.311-2.015] (p<0.001)**	**1.572 [1.264-1.956] (p<0.001)**
Age	**1.062 [1.053-1.071] (p<0.001)**	**1.067 [1.057-1.077] (p<0.001)**	**1.062 [1.053-1.071] (p<0.001)**	**1.066 [1.056-1.077] (p<0.001)**	**1.067 [1.057-1.078] (p<0.001)**
CVD	.	0.723 [0.462-1.131] (p=0.16)	.	0.751 [0.480-1.176] (p=0.21)	0.735 [0.470-1.151] (p=0.18)
T2D	.	1.040 [0.688-1.572] (p=0.85)	.	1.121 [0.748-1.681] (p=0.58)	1.047 [0.693-1.583] (p=0.83)
Hyperlipidemia	.	0.874 [0.594-1.285] (p=0.49)	.	0.857 [0.583-1.259] (p=0.43)	0.871 [0.593-1.280] (p=0.48)
Hypertension	.	0.882 [0.693-1.122] (p=0.31)	.	0.900 [0.707-1.147] (p=0.40)	0.894 [0.701-1.139] (p=0.36)

BMI	.	1.006 [0.982-1.030] (p=0.64)	.	1.011 [0.987-1.035] (p=0.38)	1.006 [0.982-1.031] (p=0.61)
PhenoAgeAcc	1.015 [0.996-1.033] (p=0.12)	1.017 [0.996-1.038] (p=0.11)	.	.	1.019 [0.998-1.040] (p=0.08)
BloodAgeAcc	.	.	0.995 [0.980-1.011] (p=0.57)	0.995 [0.978-1.012] (p=0.53)	0.992 [0.975-1.009] (p=0.34)

c)

Variable	MODEL 1 - PhenoAge HR [95% CI] (*p* value)	MODEL 2 – PhenoAge HR [95% CI] (*p* value)	MODEL 1 - BloodAge HR [95% CI] (*p* value)	MODEL 2 – BloodAge HR [95% CI] (*p* value)	MODEL 3 HR [95% CI] (*p* value)
Sex (men)	**3.198 [2.315-4.420] (p<0.001)**	**3.121 [2.228-4.371] (p<0.001)**	**3.523 [2.557-4.853] (p<0.001)**	**3.395 [2.431-4.741] (p<0.001)**	**3.121 [2.228-4.371] (p<0.001)**
Age	**1.072 [1.059-1.084] (p<0.001)**	**1.076 [1.062-1.090] (p<0.001)**	**1.071 [1.058-1.084] (p<0.001)**	**1.074 [1.060-1.088] (p<0.001)**	**1.076 [1.062-1.090] (p<0.001)**
CVD	.	1.068 [0.657-1.737] (p=0.79)	.	1.119 [0.687-1.823] (p=0.65)	1.062 [0.651-1.733] (p=0.81)
T2D	.	1.178 [0.733-1.894] (p=0.50)	.	1.409 [0.889-2.235] (p=0.14)	1.177 [0.732-1.891] (p=0.50)
Hyperlipidemia	.	1.207 [0.765-1.904] (p=0.42)	.	1.160 [0.734-1.832] (p=0.52)	1.208 [0.766-1.906] (p=0.42)
Hypertension	.	**0.710 [0.513-0.983] (p=0.039)**	.	0.725 [0.523-1.004] (p=0.05)	**0.707 [0.510-0.981] (p=0.038)**
BMI	.	1.004 [0.972-1.038] (p=0.80)	.	1.014 [0.981-1.048] (p=0.41)	1.004 [0.971-1.038] (p=0.81)
PhenoAgeAcc	**1.050 [1.029-1.071] (p<0.001)**	**1.046 [1.023-1.069] (p<0.001)**	.	.	**1.046 [1.022-1.070] (p<0.001)**
BloodAgeAcc	.	.	1.017 [0.996-1.039] (p=0.12)	1.011 [0.988-1.034] (p=0.36)	1.002 [0.980-1.026] (p=0.84)

d)

Variable	MODEL 1 - PhenoAge HR [95% CI] (*p* value)	MODEL 2 – PhenoAge HR [95% CI] (*p* value)	MODEL 1 - BloodAge HR [95% CI] (*p* value)	MODEL 2 – BloodAge HR [95% CI] (*p* value)	MODEL 3 HR [95% CI] (*p* value)
Sex (men)	.	.	.	.	.
Age	**1.074 [1.065-1.083] (p<0.001)**	**1.078 [1.067-1.088] (p<0.001)**	**1.074 [1.065-1.084] (p<0.001)**	**1.078 [1.067-1.088] (p<0.001)**	**1.078 [1.067-1.088] (p<0.001)**

CVD	.	**0.620 [0.414-0.926] (p=0.020)**	.	**0.642 [0.429-0.960] (p=0.031)**	**0.645 [0.431-0.965] (p=0.033)**
T2D	.	1.031 [0.710-1.497] (p=0.87)	.	1.027 [0.711-1.484] (p=0.89)	1.045 [0.719-1.519] (p=0.82)
Hyperlipidemia	.	1.221 [0.860-1.733] (p=0.26)	.	1.218 [0.859-1.726] (p=0.27)	1.213 [0.855-1.720] (p=0.28)
Hypertension	.	0.980 [0.780-1.231] (p=0.86)	.	0.998 [0.794-1.254] (p=0.98)	1.000 [0.796-1.259] (p=0.99)
BMI	.	0.996 [0.970-1.023] (p=0.79)	.	0.995 [0.969-1.022] (p=0.70)	0.996 [0.970-1.023] (p=0.77)
PhenoAgeAcc	0.984 [0.965-1.003] (p=0.10)	0.990 [0.969-1.010] (p=0.33)	.	.	0.995 [0.973-1.016] (p=0.61)
BloodAgeAcc	.	.	**0.980 [0.964-0.995] (p=0.009)**	**0.982 [0.966-0.998] (p=0.030)**	**0.983 [0.967-1.000]* (p=0.046)**

* [Model 3: the actual superior limit of the HR confidence interval is 0.9996]

e)

Variable	MODEL 1 - PhenoAge HR [95% CI] (*p* value)	MODEL 2 – PhenoAge HR [95% CI] (*p* value)	MODEL 1 - BloodAge HR [95% CI] (*p* value)	MODEL 2 – BloodAge HR [95% CI] (*p* value)	MODEL 3 HR [95% CI] (*p* value)
Sex (men)	1.472 [0.951-2.277] (p=0.083)	1.485 [0.947-2.330] (p=0.085)	**1.570 [1.020-2.414] (p=0.040)**	**1.574 [1.008-2.456] (p=0.046)**	1.486 [0.945-2.334] (p=0.09)
Age	**1.059 [1.040-1.079] (p<0.001)**	**1.060 [1.038-1.081] (p<0.001)**	**1.058 [1.039-1.077] (p<0.001)**	**1.059 [1.038-1.080] (p<0.001)**	**1.060 [1.039-1.081] (p<0.001)**
CVD	.	1.164 [0.536-2.529] (p=0.70)	.	1.108 [0.508-2.417] (p=0.80)	1.066 [0.487-2.329] (p=0.87)
T2D	.	1.213 [0.557-2.640] (p=0.63)	.	1.330 [0.624-2.835] (p=0.46)	1.186 [0.546-2.573] (p=0.67)
Hyperlipidemia	.	1.006 [0.485-2.088] (p=0.99)	.	0.992 [0.477-2.062] (p=0.98)	1.017 [0.490-2.115] (p=0.96)
Hypertension	.	0.964 [0.584-1.589] (p=0.88)	.	0.911 [0.551-1.507] (p=0.72)	0.900 [0.543-1.489] (p=0.68)
BMI	.	0.985 [0.937-1.035] (p=0.55)	.	0.989 [0.942-1.038] (p=0.66)	0.983 [0.935-1.032] (p=0.49)

PhenoAgeAcc	**1.040 [1.005-1.076] (p=0.025)**	**1.039 [1.002-1.078] (p=0.040)**	.	.	1.030 [0.991-1.071] (p=0.14)
BloodAgeAcc	.	.	**1.046 [1.011-1.081] (p=0.009)**	**1.047 [1.011-1.084] (p=0.009)**	**1.042 [1.005-1.079] (p=0.024)**

f)

Variable	MODEL 1 - PhenoAge HR [95% CI] (*p* value)	MODEL 2 – PhenoAge HR [95% CI] (*p* value)	MODEL 1 - BloodAge HR [95% CI] (*p* value)	MODEL 2 – BloodAge HR [95% CI] (*p* value)	MODEL 3 HR [95% CI] (*p* value)
Sex (men)	**2.341 [1.499-3.659] (p<0.001)**	**2.621 [1.635-4.204] (p<0.001)**	**2.553 [1.641-3.973] (p<0.001)**	**2.870 [1.797-4.585] (p<0.001)**	**2.623 [1.635-4.207] (p<0.001)**
Age	**1.039 [1.021-1.057] (p<0.001)**	**1.035 [1.014-1.056] (p=0.001)**	**1.039 [1.021-1.056] (p<0.001)**	**1.033 [1.013-1.054] (p=0.001)**	**1.035 [1.014-1.056] (p=0.001)**
CVD	.	0.535 [0.215-1.329] (p=0.18)	.	0.557 [0.223-1.389] (p=0.21)	0.530 [0.212-1.323] (p=0.17)
T2D	.	0.446 [0.157-1.264] (p=0.13)	.	0.549 [0.197-1.531] (p=0.25)	0.445 [0.157-1.261] (p=0.13)
Hyperlipidemia	.	1.815 [0.945-3.486] (p=0.07)	.	1.754 [0.912-3.374] (p=0.09)	1.817 [0.946-3.490] (p=0.07)
Hypertension	.	1.468 [0.902-2.389] (p=0.12)	.	1.495 [0.917-2.436] (p=0.11)	1.461 [0.894-2.385] (p=0.13)
BMI	.	1.021 [0.974-1.070] (p=0.38)	.	1.032 [0.985-1.082] (p=0.18)	1.021 [0.974-1.070] (p=0.39)
PhenoAgeAcc	**1.053 [1.023-1.084] (p<0.001)**	**1.052 [1.018-1.089] (p=0.003)**	.	.	**1.052 [1.016-1.089] (p=0.004)**
BloodAgeAcc	.	.	1.032 [0.999-1.066] (p=0.06)	1.013 [0.979-1.048] (p=0.47)	1.003 [0.969-1.039] (p=0.85)

Hazard Ratios (HRs) with 95% Confidence Intervals (CIs) for specific fatal/nonfatal **a)** breast, **b)** colorectal, **c)** lung, **d)** prostate, **e)** pancreatic and **f)** renal cancer risks are are reported for all the covariates included in incrementally adjusted Cox PH models. Median (IQR) follow-up time (incident fatal/nonfatal cancers risks): 13.12 (2.11) years. Legend: Model 1-PhenoAge: age + sex + PhenoAgeAcc; Model 2-PhenoAge: Model 1 + prevalent health conditions (CVD, T2D, hyperlipidemia, hypertension, BMI); Model 1-BloodAge: age + sex + BloodAgeAcc; Model 2- BloodAge: Model 1 + prevalent health conditions (CVD, T2D, hyperlipidemia, hypertension, BMI); Model 3: age + sex + prevalent health conditions (CVD, T2D, hyperlipidemia, hypertension, BMI) + PhenoAgeAcc + BloodAgeAcc. P values are rounded to two decimal places unless significant. Abbreviations: CVD: cardiovascular disease; T2D: type 2 diabetes; BMI: body mass index; PhenoAgeAcc: ΔPhenoAge regression residuals vs chronological age; BloodAgeAcc: ΔBloodAge regression residuals vs chronological age.

SUPPLEMENTARY DATA

Table S7. Associations between aging categories and incident fatal/nonfatal **a)** colorectal, **b)** lung, **c)** pancreatic and **d)** renal cancer risks.

a)

Variable	HR	HRinf	HRsup	P value
Sex (men)	**1.59**	**1.28**	**1.98**	**<0.001**
Age	**1.07**	**1.06**	**1.08**	**<0.001**
CVD	0.74	0.47	1.16	0.19
T2D	1.09	0.72	1.65	0.68
Hyperlipidemia	0.86	0.59	1.27	0.46
Hypertension	0.90	0.70	1.14	0.38
BMI	1.01	0.98	1.03	0.50
PhenoAgeAcc ≥ 5 y	1.12	0.82	1.51	0.48
PhenoAgeAcc ≤ -5 y	0.90	0.66	1.23	0.52
BloodAgeAcc ≥ 5 y	0.98	0.74	1.29	0.87
BloodAgeAcc ≤ -5 y	1.13	0.88	1.46	0.34

b)

Variable	HR	HRinf	HRsup	P value
Sex (men)	**3.11**	**2.22**	**4.35**	**<0.001**
Age	**1.08**	**1.06**	**1.09**	**<0.001**
CVD	1.07	0.65	1.74	0.80
T2D	1.23	0.77	1.96	0.39
Hyperlipidemia	1.21	0.77	1.92	0.41
Hypertension	**0.70**	**0.51**	**0.97**	**0.034**
BMI	1.00	0.97	1.04	0.84
PhenoAgeAcc ≥ 5 y	**1.59**	**1.12**	**2.26**	**0.010**
PhenoAgeAcc ≤ -5 y	**0.56**	**0.33**	**0.97**	**0.038**
BloodAgeAcc ≥ 5 y	1.07	0.75	1.52	0.70
BloodAgeAcc ≤ -5 y	0.93	0.64	1.35	0.70

c)

Variable	HR	HRinf	HRsup	P value
Sex (men)	1.52	0.96	2.39	0.07
Age	**1.06**	**1.04**	**1.08**	**<0.001**
CVD	1.07	0.49	2.34	0.86
T2D	1.16	0.54	2.50	0.71
Hyperlipidemia	1.02	0.49	2.13	0.95
Hypertension	0.90	0.54	1.48	0.67
BMI	0.98	0.94	1.03	0.50
PhenoAgeAcc ≥ 5 y	1.66	0.96	2.87	0.07
PhenoAgeAcc ≤ -5 y	1.00	0.51	1.94	0.99

© 2025. Morelli M et al. Published online at http://www.aginganddisease.org/EN/10.14336/AD.2025.0204

BloodAgeAcc ≥ 5 y	1.29	0.78	2.11	0.32
BloodAgeAcc ≤ -5 y	**0.40**	**0.19**	**0.86**	**0.018**

d)

Variable	HR	HRinf	HRsup	P value
Sex (men)	**2.70**	**1.68**	**4.34**	**<0.001**
Age	**1.04**	**1.02**	**1.06**	**<0.001**
CVD	0.55	0.22	1.36	0.20
T2D	0.47	0.17	1.31	0.15
Hyperlipidemia	1.82	0.94	3.49	0.07
Hypertension	1.48	0.91	2.41	0.12
BMI	1.02	0.98	1.07	0.34
PhenoAgeAcc ≥ 5 y	**1.95**	**1.17**	**3.25**	**0.011**
PhenoAgeAcc ≤ -5 y	0.89	0.43	1.83	0.75
BloodAgeAcc ≥ 5 y	0.84	0.49	1.46	0.54
BloodAgeAcc ≤ -5 y	0.78	0.43	1.39	0.39

Hazard Ratios (HRs) with 95% Confidence Intervals (CIs) for specific fatal/nonfatal **a)** colorectal, **b)** lung, **c)** pancreatic and **d)** renal cancer risks are reported for all the covariates included in incrementally adjusted Cox PH models. P values are rounded to two decimal places unless significant. Abbreviations: CVD: cardiovascular disease; T2D: type 2 diabetes; BMI: body mass index; HR: Hazard Ratio; HRinf/HRsup: Lower and Upper limit of the HR confidence interval; PhenoAgeAcc: ΔPhenoAge regression residuals vs chronological age; BloodAgeAcc: ΔBloodAge regression residuals vs chronological age.

SUPPLEMENTARY DATA

Table S8. Associations between aging clocks tested and incident cancer-related risks, excluding subjects with events occurring in the first year of follow-up.

Aging Clock	MODEL 3<br>HR [95% CI]<br>(*p* value)	Cancer Event
PhenoAgeAcc	**1.042 [1.030-1.055] (p<0.001)**	**Death**
BloodAgeAcc	**1.013 [1.001-1.024] (p=0.028)**	
PhenoAgeAcc	**1.042 [1.023-1.040] (p<0.001)**	**First Hospitalization**
BloodAgeAcc	1.013 [0.989-1.003] (p=0.30)	
PhenoAgeAcc	1.004 [0.978-1.030] (p=0.77)	BC
BloodAgeAcc	0.979 [0.958-1.000] (p=0.05)	
PhenoAgeAcc	1.013 [0.990-1.036] (p=0.28)	CRC
BloodAgeAcc	0.990 [0.972-1.008] (p=0.28)	
PhenoAgeAcc	**1.044 [1.020-1.069] (p<0.001)**	**LC**
BloodAgeAcc	1.003 [0.980-1.026] (p=0.82)	
PhenoAgeAcc	0.993 [0.971-1.015] (p=0.51)	**PC**
BloodAgeAcc	**0.981 [0.965-0.999] (p=0.035)**	
PhenoAgeAcc	1.029 [0.987-1.073] (p=0.18)	**PNC**
BloodAgeAcc	**1.041 [1.003-1.080] (p=0.033)**	
PhenoAgeAcc	**1.051 [1.014-1.089] (p=0.007)**	**RNC**
BloodAgeAcc	1.002 [0.967-1.038] (p=0.91)	

Risk estimates for cancer deaths, first cancer hospitalizations and specific fatal/nonfatal (breast, colorectal, lung, prostate, pancreatic and renal) cancer risks are expressed as Hazard Ratios (HRs) with 95% Confidence Intervals (CIs) for annual increase in Δage, as calculated through Cox PH incrementally adjusted multivariable models. P values are rounded to two decimal places unless significant. Significant associations (p<0.05) are highlighted in bold.
Legend: Model 3: age + sex + prevalent health conditions (CVD, T2D, hyperlipidemia, hypertension, BMI + PhenoAgeAcc + BloodAgeAcc.
Abbreviations: CVD: cardiovascular disease; T2D: type 2 diabetes; BMI: body mass index; BC: breast cancer; CRC: colorectal cancer; LC: lung cancer; PC: prostate cancer, PNC: pancreatic cancer; RNC: renal cancer; PhenoAgeAcc: ΔPhenoAge regression residuals vs chronological age; BloodAgeAcc: ΔBloodAge regression residuals vs chronological age.

© 2025. Morelli M et al. Published online at http://www.aginganddisease.org/EN/10.14336/AD.2025.0204

SUPPLEMENTARY DATA

Table S9. Associations between tested aging clocks and risk of incident cancer-related events.

Aging Clock	MODEL 4 - PhenoAge HR [95% CI] (*p* value)	MODEL 4 – BloodAge HR [95% CI] (*p* value)	MODEL 5 HR [95% CI] (*p* value)	Cancer Event
PhenoAgeAcc	**1.037 [1.025-1.050] (p<0.001)**	.	**1.035 [1.022-1.048] (p<0.001)**	Death
BloodAgeAcc	.	**1.017 [1.006-1.029] (p=0.002)**	**1.012 [1.000-1.023] (p=0.042)**	
PhenoAgeAcc	**1.028 [1.020-1.036] (p<0.001)**	.	**1.029 [1.021-1.037] (p<0.001)**	First Hospitalization
BloodAgeAcc	.	1.000 [0.993-1.007] (p=0.95)	0.996 [0.989-1.003] (p=0.23)	
PhenoAgeAcc	0.994 [0.969-1.020] (p=0.64)	.	0.997 [0.972-1.023] (p=0.84)	BC
BloodAgeAcc	.	**0.976 [0.956-0.997] (p=0.023)**	**0.977 [0.956-0.997] (p=0.025)**	
PhenoAgeAcc	1.017 [0.996-1.038] (p=0.11)	.	1.019 [0.998-1.040] (p=0.08)	CRC
BloodAgeAcc	.	0.995 [0.978-1.013] (p=0.60)	0.993 [0.976-1.010] (p=0.41)	
PhenoAgeAcc	**1.029 [1.003-1.055] (p=0.027)**	.	**1.030 [1.004-1.056] (p=0.026)**	LC
BloodAgeAcc	.	1.002 [0.979-1.025] (p=0.89)	0.997 [0.974-1.020] (p=0.77)	
PhenoAgeAcc	0.991 [0.970-1.012] (p=0.40)	.	0.995 [0.974-1.017] (p=0.66)	PC
BloodAgeAcc	.	**0.983 [0.967-0.999] (p=0.042)**	0.984 [0.968-1.000] (p=0.06)	
PhenoAgeAcc	1.036 [0.997-1.077] (p=0.07)	.	1.027 [0.987-1.069] (p=0.19)	PNC
BloodAgeAcc	.	**1.048 [1.011-1.085] (p=0.009)**	**1.043 [1.006-1.081] (p=0.021)**	
PhenoAgeAcc	**1.046 [1.011-1.084] (p=0.011)**	.	**1.046 [1.009-1.083] (p=0.014)**	RNC
BloodAgeAcc	.	1.012 [0.978-1.048] (p=0.49)	1.005 [0.970-1.040] (p=0.79)	

Risk estimates for (a) cancer deaths, (b) first cancer hospitalizations and (c) specific fatal/nonfatal (breast, colorectal, lung, prostate, pancreatic and renal) cancer risks are expressed as Hazard Ratios (HRs) with 95% Confidence Intervals (CIs) for annual increase in Δage, as calculated through Cox PH incrementally adjusted multivariable models. P values are rounded to two decimal places unless significant. Significant associations (p<0.05) are highlighted in bold. Associations of all the covariates included in the models are reported in the Supplementary Results.
Legend: Model 4-PhenoAge: age + sex + prevalent health conditions (CVD, T2D, hyperlipidemia, hypertension, BMI) + lifestyle factors (smoking status, leisure physical activity, MDscore, education level) + PhenoAgeAcc. Model 4-BloodAge: age + sex + prevalent health conditions (CVD, T2D, hyperlipidemia, hypertension, BMI) + lifestyle factors (smoking status, leisure physical activity, MDscore, education level) + BloodAgeAcc; Model 5 : age + sex + prevalent health conditions (CVD, T2D, hyperlipidemia, hypertension, BMI) + lifestyle factors (smoking status, leisure physical activity, MDscore, education level) + PhenoAgeAcc + BloodAgeAcc.
Abbreviations: CVD: cardiovascular disease; T2D: type 2 diabetes; BMI: body mass index; MDscore: Mediterranean Diet score; BC: breast cancer; CRC: colorectal cancer; LC: lung cancer; PC: prostate cancer, PNC: pancreatic cancer; RNC: renal cancer; PhenoAgeAcc: ΔPhenoAge regression residuals vs chronological age; BloodAgeAcc: ΔBloodAge regression residuals vs chronological age..

© 2025. Morelli M et al. Published online at http://www.aginganddisease.org/EN/10.14336/AD.2025.0204

SUPPLEMENTARY DATA

Table S10. Interaction analyses of the aging clocks tested with covariates.

a)

**Aging Clock**	**Interaction with**	**HR [95% CI]<br>(*p* value)**	**Cancer Event**
PhenoAgeAcc	Sex (men vs women)	0.995 [0.970-1.020]<br>(p = 0.67)	Cancer Death
BloodAgeAcc	Sex (men vs women)	1.008 [0.986-1.032]<br>(p = 0.48)	Cancer Death
PhenoAgeAcc	Age	1.000 [0.999-1.001]<br>(p = 0.65)	Cancer Death
BloodAgeAcc	Age	1.000 [0.999-1.001]<br>(p = 0.89)	Cancer Death
PhenoAgeAcc	Education<br>(lower secondary vs primary/lower)	0.984 [0.956-1.013]<br>(p = 0.28)	Cancer Death
PhenoAgeAcc	Education<br>(upper secondary)	0.990 [0.963-1.018]<br>(p = 0.49)	Cancer Death
PhenoAgeAcc	Education<br>(post-secondary)	0.965 [0.914-1.019]<br>(p = 0.20)	Cancer Death
BloodAgeAcc	Education<br>(lower secondary vs primary/lower)	1.010 [0.982-1.038]<br>(p = 0.49)	Cancer Death
BloodAgeAcc	Education<br>(upper secondary)	1.002 [0.976-1.029]<br>(p = 0.89)	Cancer Death
BloodAgeAcc	Education<br>(post-secondary)	0.999 [0.956-1.044]<br>(p = 0.98)	Cancer Death
PhenoAgeAcc	Smoking<br>(current vs never)	1.022 [0.993-1.051]<br>(p = 0.15)	Cancer Death
PhenoAgeAcc	Smoking<br>(previous vs never)	1.000 [0.973-1.028]<br>(p = 0.98)	Cancer Death
BloodAgeAcc	Smoking<br>(current vs never)	0.979 [0.951-1.006]<br>(p = 0.13)	Cancer Death
BloodAgeAcc	Smoking<br>(previous vs never)	0.995 [0.970-1.020]<br>(p = 0.66)	Cancer Death
PhenoAgeAcc	BMI	1.000 [0.998-1.003]<br>(p = 0.63)	Cancer Death
BloodAgeAcc	BMI	1.000 [0.999-1.003]<br>(p = 0.46)	Cancer Death

b)

**Aging Clock**	**Interaction with**	**HR [95% CI]<br>(*p* value)**	**Cancer Event**
PhenoAgeAcc	Sex (men vs women)	1.007 [0.992-1.022]<br>(p = 0.36)	First Hospitalization
PhenoAgeAcc	Age	1.001 [1.000-1.001]<br>(p = 0.09)	First Hospitalization
PhenoAgeAcc	Education<br>(lower secondary vs primary/lower)	0.988 [0.970-1.006]<br>(p = 0.18)	First Hospitalization
**PhenoAgeAcc**	**Education<br>(upper secondary)**	**0.975 [0.959-0.993]<br>(p = 0.005)**	**First Hospitalization**
PhenoAgeAcc	Education<br>(post-secondary)	0.978 [0.951-1.006]<br>(p = 0.12)	First Hospitalization
**PhenoAgeAcc**	**Smoking<br>(current vs never)**	**1.020 [1.001-1.038]<br>(p = 0.037)**	**First Hospitalization**
PhenoAgeAcc	Smoking<br>(previous vs never)	1.015 [0.998-1.033]<br>(p = 0.08)	First Hospitalization

© 2025. Morelli M et al. Published online at http://www.aginganddisease.org/EN/10.14336/AD.2025.0204

c)

**Aging Clock**	**Interaction with**	**HR [95% CI] (*p* value)**	**Cancer Event**
BloodAgeAcc	Age	1.001 [1.000-1.004] (p = 0.05)	BC
BloodAgeAcc	Education (lower secondary vs primary/lower)	0.968 [0.916-1.022] (p = 0.24)	BC
BloodAgeAcc	Education (upper secondary)	0.958 [0.912-1.006] (p = 0.08)	BC
BloodAgeAcc	Education (post-secondary)	0.955 [0.890-1.025] (p = 0.20)	BC
BloodAgeAcc	BMI	1.003 [0.999-1.006] (p = 0.10)	BC

d)

**Aging Clock**	**Interaction with**	**HR [95% CI] (*p* value)**	**Cancer Event**
PhenoAgeAcc	Sex (men vs women)	1.023 [0.962-1.088] (p = 0.46)	LC
PhenoAgeAcc	Age	1.000 [0.998-1.002] (p = 0.96)	LC
PhenoAgeAcc	Smoking (current vs never)	1.072 [0.998-1.152] (p = 0.06)	LC
PhenoAgeAcc	Smoking (previous vs never)	1.021 [0.946-1.102] (p = 0.59)	LC

e)

**Aging Clock**	**Interaction with**	**HR [95% CI] (*p* value)**	**Cancer Event**
BloodAgeAcc	Age	0.999 [0.996-1.002] (p = 0.44)	PNC

f)

**Aging Clock**	**Interaction with**	**HR [95% CI] (*p* value)**	**Cancer Event**
PhenoAgeAcc	Sex (men vs women)	1.077 [0.984-1.178] (p = 0.11)	RNC
PhenoAgeAcc	Age	0.999 [0.996-1.002] (p = 0.45)	RNC
PhenoAgeAcc	Education (lower secondary vs primary/lower)	1.010 [0.922-1.107] (p = 0.82)	RNC
PhenoAgeAcc	Education (upper secondary)	0.996 [0.898-1.105] (p = 0.94)	RNC
PhenoAgeAcc	Education (post-secondary)	1.035 [0.914-1.173] (p = 0.59)	RNC
PhenoAgeAcc	Smoking (current vs never)	1.028 [0.918-1.152] (p = 0.63)	RNC
PhenoAgeAcc	Smoking (previous vs never)	1.066 [0.972-1.170] (p = 0.18)	RNC

SUPPLEMENTARY DATA

Hazard Ratios (HRs) with 95% Confidence Intervals (CIs) and relevant p-values are report for those incident events, clocks and covariates which showed at least a nominally significant association within the lifestyle-enriched model. Associations with **a)** Cancer deaths, **b)** first hospitalization, fatal/nonfatal **c)** breast (BC), **d)** lung (LC), **e)** pancreatic (PNC) and **f)** renal cancer (RNC) are reported. Abbreviations: BMI: body mass index; PhenoAgeAcc: ΔPhenoAge regression residuals vs chronological age; BloodAgeAcc: ΔBloodAge regression residuals vs chronological age.

Table S11. Association of incident prostate cancer risk vs BloodAgeAcc obtained from a linear regression against glucose levels.

**Variable**	**HR**	**HRinf**	**HRsup**	**P value**
Age	**1.08**	**1.07**	**1.09**	**<0.001**
CVD	**0.64**	**0.43**	**0.96**	**0.032**
T2D	1.02	0.70	1.48	0.93
Hyperlipidemia	1.21	0.85	1.72	0.28
Hypertension	1.00	0.80	1.26	1.00
BMI	1.00	0.97	1.02	0.75
PhenoAgeAcc	0.99	0.97	1.01	0.54
BloodAgeAcc_GlucRes	0.98	0.97	1.00	0.06

Abbreviations: CVD: cardiovascular disease; T2D: type 2 diabetes; BMI: body mass index; HR: Hazard Ratio; HRinf/HRsup: Lower and Upper limit of the HR confidence interval; PhenoAgeAcc: ΔPhenoAge regression residuals vs chronological age; BloodAgeAcc: ΔBloodAge regression residuals vs chronological age; BloodAgeAcc_GlucRes: linear regression residuals of BloodAgeAcc vs circulating glucose levels.

© 2025. Morelli M et al. Published online at http://www.aginganddisease.org/EN/10.14336/AD.2025.0204

SUPPLEMENTARY DATA

Table S12. Interaction analysis between BloodAgeAcc and family history of prostate cancer.

Variable	HR	HRinf	HRsup	P value
Age	**1.08**	**1.07**	**1.09**	**<0001**
CVD	**0.65**	**0.43**	**0.97**	**0.036**
T2D	1.04	0.71	1.51	0.85
Hyperlipidemia	1.21	0.85	1.71	0.29
Hypertension	1.00	0.79	1.25	0.97
BMI	1.00	0.97	1.02	0.78
ΔPhenoAge	0.99	0.97	1.02	0.61
ΔBloodAge	0.98	0.97	1.00	0.05
PC Familiarity	1.62	0.99	2.66	0.06
BloodAgeAcc * PC Familiarity	1.00	0.92	1.08	0.96

Associations with incident prostate cancer risk are reported as Hazard Ratios (HRs) with 95% Confidence Intervals (CIs) and relevant p-values, are reported for all the covariates included in Model 3 and an additional interaction term between BloodAgeAcc and family history of prostate cancer (PC Familiarity). Abbreviations: CVD: cardiovascular disease; T2D: type 2 diabetes; BMI: body mass index; HR: Hazard Ratio; HRinf/HRsup: Lower and Upper limit of the HR confidence interval; BloodAgeAcc: ΔBloodAge regression residuals vs chronological age.

© 2025. Morelli M et al. Published online at http://www.aginganddisease.org/EN/10.14336/AD.2025.0204

SUPPLEMENTARY DATA

Table S13. Interaction analysis between BloodAgeAcc and several risk/protective factors for breast cancer.

a)

Variable	HR	HRinf	HRsup	P value
Age	1.01	1.00	1.02	0.20
CVD	1.26	0.72	2.22	0.41
T2D	1.01	0.55	1.86	0.98
Hyperlipidemia	1.13	0.74	1.74	0.56
Hypertension	0.98	0.73	1.31	0.87
BMI	**1.02**	**1.00**	**1.05**	**0.040**
ΔPhenoAge	1.01	0.98	1.03	0.62
ΔBloodAge	**0.97**	**0.95**	**0.99**	**0.011**
BC Familiarity	**1.08**	**1.21**	**2.68**	**0.004**
BloodAgeAcc * BC Familiarity	1.07	1.00	1.15	0.05

b)

Variable	HR	HRinf	HRsup	P value
Age	1.01	0.99	1.03	0.25
CVD	1.22	0.69	2.14	0.49
T2D	0.99	0.54	1.83	0.98
Hyperlipidemia	1.16	0.75	1.77	0.51
Hypertension	0.97	0.72	1.30	0.82
BMI	**1.03**	**1.00**	**1.05**	**0.027**
PhenoAgeAcc	1.01	0.98	1.03	0.62
BloodAgeAcc	**0.95**	**0.91**	**0.99**	**0.008**
Menopause	0.99	0.68	1.45	0.97
BloodAgeAcc * Menopause	1.05	1.00	1.09	0.06

© 2025. Morelli M et al. Published online at http://www.aginganddisease.org/EN/10.14336/AD.2025.0204

c)

Variable	HR	HRinf	HRsup	P value
Age	1.01	1.00	1.02	0.15
CVD	1.30	0.74	2.28	0.37
T2D	1.06	0.58	1.96	0.84
Hyperlipidemia	1.11	0.72	1.71	0.64
Hypertension	0.99	0.73	1.33	0.92
BMI	**1.03**	**1.00**	**1.05**	**0.031**
PhenoAgeAcc	1.00	0.98	1.03	0.92
BloodAgeAcc	**0.98**	**0.96**	**1.00**	**0.036**
Early Menopause	0.79	0.51	1.21	0.28
BloodAgeAcc * Early Menopause	1.03	0.96	1.10	0.42

d)

Variable	HR	HRinf	HRsup	P value
Age	1.01	1.00	1.02	0.19
CVD	1.21	0.67	2.16	0.53
T2D	1.08	0.58	1.99	0.81
Hyperlipidemia	1.14	0.74	1.75	0.56
Hypertension	0.99	0.73	1.34	0.95
BMI	1.02	1.00	1.05	0.08
PhenoAgeAcc	1.00	0.97	1.03	0.99
BloodAgeAcc	**0.98**	**0.95**	**1.00**	**0.044**
Early Menarche	1.08	0.82	1.43	0.58
BloodAgeAcc * Early Menarche	1.03	0.98	1.08	0.23

e)

Variable	HR	HRinf	HRsup	P value
Age	1.01	1.00	1.02	0.16
CVD	1.24	0.71	2.17	0.46
T2D	1.01	0.55	1.87	0.96
Hyperlipidemia	1.14	0.74	1.74	0.55
Hypertension	0.98	0.73	1.31	0.88
BMI	**1.03**	**1.00**	**1.05**	**0.038**
PhenoAgeAcc	1.01	0.98	1.03	0.60
BloodAgeAcc	1.03	0.97	1.09	0.38
Parity	0.95	0.64	1.40	0.78
BloodAgeAcc * Parity	0.95	0.89	1.01	0.09

Associations with incident breast cancer risk are reported as Hazard Ratios (HRs) with 95% Confidence Intervals (CIs) and relevant p-values, for all the covariates included in Model 3 and additional interaction terms between BloodAgeAcc and **a)** family history of breast cancer (BC Familiarity), **b)** menopause (yes vs no), **c)** early menopause (below vs above 45 years of age), **d)** early menarche (below vs above 12 years), and **e)** parity (having no vs one or more children). P-values are rounded to two decimal places unless significant.
Abbreviations: CVD: cardiovascular disease; T2D: type 2 diabetes; BMI: body mass index; HR: Hazard Ratio; HRinf/HRsup: Lower and Upper limit of the HR confidence interval; PhenoAgeAcc: ΔPhenoAge regression residuals vs chronological age; BloodAgeAcc: ΔBloodAge regression residuals vs chronological age.

© 2025. Morelli M et al. Published online at http://www.aginganddisease.org/EN/10.14336/AD.2025.0204

SUPPLEMENTARY DATA

Table S14. Associations between incident breast cancer risk and BloodAgeAcc stratified by breast cancer familiarity.

a)

Variable	HR	HRinf	HRsup	P value
Age	1.02	0.98	1.07	0.27
CVD	0.75	0.09	5.97	0.78
T2D	1.01	0.21	4.84	0.99
Hyperlipidemia	0.97	0.27	3.55	0.97
Hypertension	1.15	0.46	2.87	0.76
BMI	**1.09**	**1.03**	**1.16**	**0.005**
PhenoAgeAcc	1.00	0.92	1.09	1.00
BloodAgeAcc	1.02	0.95	1.09	0.66

b)

Variable	HR	HRinf	HRsup	P value
Age	1.01	0.99	1.02	0.28
CVD	1.33	0.74	2.39	0.34
T2D	0.98	0.50	1.91	0.96
Hyperlipidemia	1.16	0.74	1.83	0.51
Hypertension	0.96	0.70	1.31	0.80
BMI	1.02	0.99	1.04	0.24
PhenoAgeAcc	1.01	0.98	1.03	0.56
BloodAgeAcc	**0.97**	**0.95**	**1.00**	**0.019**

Associations with incident breast cancer - expressed as Hazard Ratios (HRs) with 95% Confidence Intervals (CIs) and relevant p-values - are reported for ΔBloodAge and other covariates in subjects reporting **a)** family (N = 625) and **b)** no family history of breast cancer cases (N = 11,147)**.** P-values are rounded to two decimal places unless significant. Abbreviations: CVD: cardiovascular disease; T2D: type 2 diabetes; BMI: body mass index; HR: Hazard Ratio; HRinf/HRsup: Lower and Upper limit of the HR confidence interval; PhenoAgeAcc: ΔPhenoAge regression residuals vs chronological age; BloodAgeAcc: ΔBloodAge regression residuals vs chronological age.

Table S15. Associations between incident breast cancer risk and BloodAgeAcc stratified by breast cancer molecular subtype.

a)

Variable	HR	HRinf	HRsup	P value
Age	1.01	1.00	1.02	0.14
CVD	1.24	0.69	2.23	0.46
T2D	0.88	0.45	1.71	0.70
Hyperlipidemia	1.15	0.74	1.79	0.52
Hypertension	0.97	0.72	1.32	0.85
BMI	1.02	1.00	1.05	0.07
PhenoAgeAcc	1.01	0.99	1.04	0.38
BloodAgeAcc	**0.98**	**0.96**	**1.00**	**0.041**

b)

Variable	HR	HRinf	HRsup	P value
Age	**1.03**	**1.01**	**1.04**	**0.006**
CVD	1.03	0.41	2.62	0.94
T2D	1.52	0.67	3.46	0.31
Hyperlipidemia	0.80	0.39	1.65	0.55
Hypertension	0.85	0.54	1.33	0.48

BMI	1.02	0.99	1.06	0.24
PhenoAgeAcc	1.01	0.97	1.05	0.66
BloodAgeAcc	0.98	0.95	1.01	0.18

c)

Variable	HR	HRinf	HRsup	P value
Age	1.01	1.00	1.02	0.16
CVD	1.24	0.68	2.28	0.48
T2D	0.94	0.48	1.83	0.85
Hyperlipidemia	1.16	0.73	1.84	0.52
Hypertension	0.92	0.67	1.27	0.61
BMI	1.02	0.99	1.05	0.12
PhenoAgeAcc	1.02	0.99	1.04	0.23
BloodAgeAcc	0.98	0.96	1.01	0.17

d)

Variable	HR	HRinf	HRsup	P value
Age	**1.02**	**1.01**	**1.04**	**0.007**
CVD	1.07	0.46	2.50	0.88
T2D	1.32	0.59	2.97	0.50
Hyperlipidemia	0.84	0.44	1.61	0.59
Hypertension	0.95	0.63	1.44	0.80
BMI	1.03	0.99	1.06	0.13
PhenoAgeAcc	1.00	0.96	1.04	0.99
BloodAgeAcc	**0.97**	**0.94**	**1.00**	**0.023**

e)

Variable	HR	HRinf	HRsup	P value
Age	1.01	1.00	1.03	0.08
CVD	1.53	0.73	3.24	0.26
T2D	1.28	0.57	2.88	0.54
Hyperlipidemia	0.63	0.31	1.29	0.21
Hypertension	1.01	0.67	1.52	0.96
BMI	1.02	0.98	1.05	0.34
PhenoAgeAcc	1.01	0.97	1.04	0.68
BloodAgeAcc	0.98	0.95	1.01	0.13

f)

Variable	HR	HRinf	HRsup	P value
Age	**1.01**	**1.00**	**1.03**	**0.046**
CVD	1.05	0.56	1.96	0.89
T2D	0.90	0.46	1.75	0.76
Hyperlipidemia	1.21	0.78	1.88	0.39
Hypertension	0.91	0.67	1.24	0.56
BMI	1.02	1.00	1.05	0.11
PhenoAgeAcc	1.01	0.99	1.04	0.33
BloodAgeAcc	0.98	0.96	1.00	0.08

© 2025. Morelli M et al. Published online at http://www.aginganddisease.org/EN/10.14336/AD.2025.0204

SUPPLEMENTARY DATA

Associations with incident breast cancer are reported as Hazard Ratios (HRs) with 95% Confidence Intervals (CIs) and relevant p-values, for BloodAgeAcc and other covariates in Cox PH regression models stratified by estrogen receptor (ER) type (**a)** positive (N = 188) vs **b)** negative (N = 21); progesterone receptor (PGR) type (**c)** positive (N = 168) vs **d)** negative (N = 41) and human epidermal growth factor receptor 2 (HER2) type (**e)** positive (N = 29) vs **f)** negative (N = 154). P-values are rounded to two decimal places unless significant.
Abbreviations: CVD: cardiovascular disease; T2D: type 2 diabetes; BMI: body mass index; HR: Hazard Ratio; Hrinf/HRsup: Lower and Upper limit of the HR confidence interval; PhenoAgeAcc: ΔPhenoAge regression residuals vs chronological age; BloodAgeAcc: ΔBloodAge regression residuals vs chronological age.

Moli-sani Study Investigators
The enrolment phase of the Moli-sani Study was conducted at the Research Laboratories of the Catholic University in Campobasso (Italy), the follow up of the Moli-sani cohort is being conducted at the Department of Epidemiology and Prevention of the IRCCS Neuromed, Pozzilli, Italy.
Steering Committee: Licia Iacoviello*[#] (chairperson), Giovanni de Gaetano* and Maria Benedetta Donati*.
Scientific Secretariat: Chiara Cerletti* (coordinator), Marialaura Bonaccio*, Americo Bonanni*, Simona Costanzo*°, Amalia De Curtis*, Augusto Di Castelnuovo*, Alessandro Gialluisi*[#], Francesco Gianfagna°[§], Mariarosaria Persichillo*, Teresa Di Prospero* (secretary).
Safety and Ethical Committee: Jos Vermylen (Catholic University, Leuven, Belgium) (Chairperson), Renzo Pegoraro (Pontificia Accademia per la Vita, Roma, Italy), Antonio Spagnolo (Catholic University, Roma, Italy).
External Event Adjudicating Committee: Deodato Assanelli (Brescia, Italy), Livia Rago (Campobasso, Italy).
Baseline and Follow-up Data Management: Simona Costanzo*° (coordinator), Marco Olivieri (Campobasso, Italy), Sabatino Orlandi*, Teresa Panzera*.
Data Analysis: Augusto Di Castelnuovo* (coordinator), Marialaura Bonaccio*, Simona Costanzo*°, Simona Esposito*, Alessandro Gialluisi*[#], Anwal Ghulam*, Francesco Gianfagna°[§], Antonietta Pepe*, Emilia Ruggiero*, Francesca Bracone*, Sukshma Sharma*.
Biobank, Molecular and Genetic Laboratory: Amalia De Curtis* (Coordinator), Concetta Civitillo*[†], Alisia Cretella*[†], Sara Magnacca*, Fabrizia Noro*.
Recruitment Staff: Mariarosaria Persichillo* (coordinator), Francesca Bracone*, Giuseppe Di Costanzo*, Fiorella De Rita (Cuore Sano ETS, Campobasso), Sabrina Franciosa*, Martina Morelli*[†], Teresa Panzera*.
Communication and Press Office: Americo Bonanni*.
Regional Institutions: Direzione Generale per la Salute - Regione Molise; Azienda Sanitaria Regionale del Molise (ASReM, Italy); Agenzia Regionale per la Protezione Ambientale del Molise (ARPA Molise, Italy); Molise Dati Spa (Campobasso, Italy); Offices of vital statistics of the Molise region.
Hospitals: Presidi Ospedalieri ASReM: Ospedale A. Cardarelli – Campobasso, Ospedale F. Veneziale – Isernia, Ospedale San Timoteo - Termoli (CB), Ospedale Ss. Rosario - Venafro (IS), Ospedale Vietri – Larino (CB), Ospedale San Francesco Caracciolo - Agnone (IS); Casa di Cura Villa Maria - Campobasso; Responsible Research Hospital - Campobasso; IRCCS Neuromed - Pozzilli (IS).
*Department of Epidemiology and Prevention, IRCCS Neuromed, Pozzilli, Italy
[#]Department of Medicine and Surgery, LUM University "Giuseppe Degennaro", Casamassima, Italy
[§]Mediterranea Cardiocentro, Napoli, Italy
°Department of Medicine and Surgery, University of Insubria, Varese, Italy
[†]Fondazione Veronesi – Piattaforma UMBERTO

Moli-sani Study Past Investigators are available at https://www.moli-sani.org/?page_id=173
